# Synergistic Evolution of Alloy Nanoparticles and Carbon in Solid-State Lithium Metal Anode Composites at Low Stack Pressure

**DOI:** 10.1021/acsnano.4c07687

**Published:** 2024-07-29

**Authors:** Sun Geun Yoon, Bairav S. Vishnugopi, Elif Pınar Alsaç, Won Joon Jeong, Stephanie Elizabeth Sandoval, Douglas Lars Nelson, Abhinand Ayyaswamy, Partha P. Mukherjee, Matthew T. McDowell

**Affiliations:** † George W. Woodruff School of Mechanical Engineering, Georgia Institute of Technology, Atlanta, Georgia 30332, United States; ‡ School of Mechanical Engineering, Purdue University, West Lafayette, Indiana 47907, United States; § School of Materials Science and Engineering, Georgia Institute of Technology, Atlanta, Georgia 30332, United States

**Keywords:** solid-state battery, energy storage, electrochemistry, lithium metal anode, battery degradation, lithium metal composite, graphene oxide

## Abstract

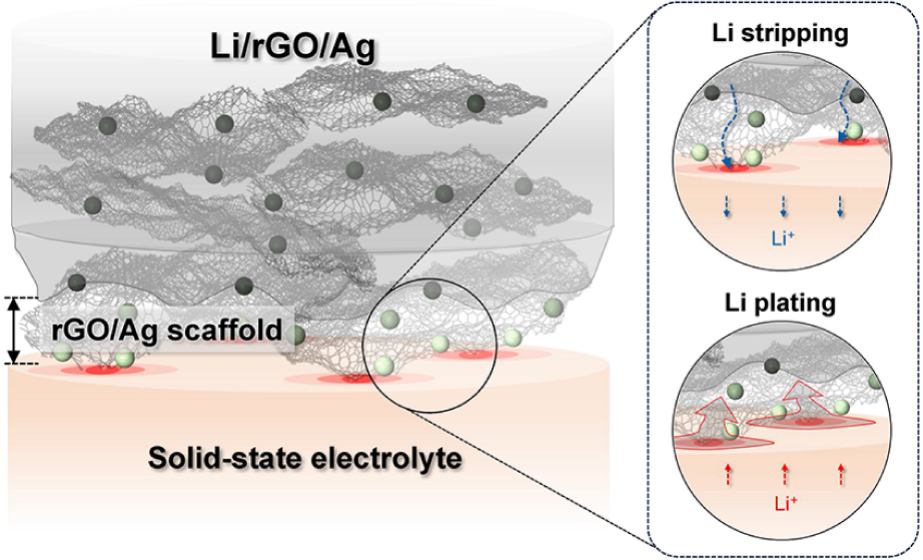

Solid-state batteries with Li metal anodes can offer increased energy density compared to Li-ion batteries. However, the performance of pure Li anodes has been limited by morphological instabilities at the interface between Li and the solid-state electrolyte (SSE). Composites of Li metal with other materials such as carbon and Li alloys have exhibited improved cycling stability, but the mechanisms associated with this enhanced performance are not clear, especially at the low stack pressures needed for practical viability. Here, we investigate the structural evolution and correlated electrochemical behavior of Li metal composites containing reduced graphene oxide (rGO) and Li–Ag alloy particles. The nanoscale carbon scaffold maintains homogeneous contact with the SSE during stripping and facilitates Li transport to the interface; these effects largely prevent interfacial disconnection even at low stack pressure. The Li–Ag is needed to ensure cyclic refilling of the rGO scaffold with Li during plating, and the solid-solution character of Li–Ag improves cycling stability compared to other materials that form intermetallic compounds. Full cells with sulfur cathodes were tested at relatively low stack pressure, achieving 100 stable cycles with 79% capacity retention.

## Introduction

Lithium metal negative electrodes can provide high specific and volumetric capacities (theoretical capacities of 3860 mAh g^–1^ and 2061 mAh cm^–3^) and therefore high energy density.^1,2^ This advantage is especially pronounced when pairing with inorganic solid-state electrolytes (SSEs), which can feature improved chemical stability and enhanced safety compared to liquid electrolytes in Li-ion batteries.^3^ However, interfacial morphological instabilities have slowed the practical application of Li metal anodes in solid-state batteries (SSBs). Although typical inorganic sulfide and oxide SSEs have higher elastic modulus (22 GPa for Li_6_PS_5_Cl (LPSC) and 175 GPa for Li_7_La_3_Zr_2_O_12_) than pure Li (6.2 GPa),^1,4^ Li-based SSBs suffer from short circuiting during cycling caused by filamentary Li growth and propagation through the SSE. Filament growth is influenced by contact loss, interfacial inhomogeneities, bulk properties of the SSE such as fracture toughness,^1,4−10^ and external factors such as temperature and stack pressure.^4,11−15^ Interfacial contact loss between the anode and SSE arising from nonuniform Li stripping is a critical issue, since it causes current constriction upon subsequent plating and leads directly to Li filament growth.^1,5,6^ Thus, engineering efforts to maintain interfacial contact are a key focus for improving Li metal SSB performance.

Exploiting the plastic deformation of Li to maintain interfacial contact has been pursued through control of temperature and stack pressure. For instance, contact can be enhanced by stripping/plating at high temperatures or through reconditioning thermal treatments, which take advantage of Li creep deformation and diffusion to retain contact.^11,12^ Applying higher stack pressure is an easily accessible approach that drives plastic deformation to ensure interfacial contact.^1,4,13,16^ However, after initiation of filaments or dendrites and spalling of the SSE interface, even a moderate level of uniaxial stack pressure encourages the filaments to propagate cracks through the SSE, highlighting the conflicting influence of stack pressure on contact retention vs filament growth.^13−15^ Furthermore, application of stack pressures beyond ∼1–2 MPa is not feasible for most commercial applications since bulky housings are needed, negating specific energy/energy density gains.^2,17,18^ Indeed, the disparity between SSB stack pressures usually used in literature reports (up to hundreds of MPa)^13,17,19^ and those attainable in practical cell designs is a critical issue in the SSB community.

Attempts to control the morphological instabilities of Li metal anodes at the SSE interface have also been made by introducing other interfacial materials, such as Li alloys^20−23^ or Li-hosting scaffolds.^24−27^ Alloy materials at the Li-SSE interface can beneficially affect both Li plating and stripping. Materials such as silver and gold form Li alloys and can reduce the nucleation overpotential for Li metal deposition, which enables homogeneous Li growth during deposition.^22,23,28^ However, in the case of intermetallic alloys, stripping can still cause interfacial disconnection, and films undergo significant structural changes due to volume changes during cycling, which can cause capacity decay by alloy agglomeration and pulverization.^19,22,29,30^ Li stripping from solid solution alloys is slightly different; Krauskopf et al. demonstrated that stripping from the β-phase Li–Mg alloy mitigated interfacial contact loss in the absence of stack pressure.^20,21,31^ Retained contact at the SSE interface during delithiation is beneficial in terms of homogenization of the stripping current distribution.^22^ However, the Li diffusion kinetics in the Li–Mg alloy was found to be a bottleneck that limited the stripping capacity.^20,21,31^

Unlike alloy interlayers, carbon scaffolds can host plated Li within their porous structures. Chen et al. demonstrated reversible Li deposition and stripping in a confined carbon nanotube scaffold.^24,32^ The hosting of Li within the carbon scaffold was thought to reduce mechanical stresses exerted on the SSE as compared to conventional plating/stripping. The boundaries between the Li metal and nanotubes also enhanced Li diffusion toward the SSE interface, thereby enabling higher stripping capacity at low stack pressures compared to pure Li.^25^ The Ag-carbon interlayer developed by Samsung combines both the alloying and carbon scaffolding approaches.^26,33^ The porous carbon scaffold decorated with Ag nanoparticles (NPs) led to Li deposition at the interface between the current collector and carbon scaffold, alleviating the risk of SSE cracking and subsequent filament growth. It was proposed that the solid solution behavior of the Li–Ag alloy could be responsible for such behavior,^33,34^ and other results have shown that chemical reactions between the Li–Ag alloy and carbon can play a role.^27^ However, the action of these various interlayer materials is quite complex, and there is not a clear understanding of the combined effects of alloys and carbon scaffolds on Li morphology reversibility, species transport, and interfacial degradation mechanisms.

**Figure 1:**
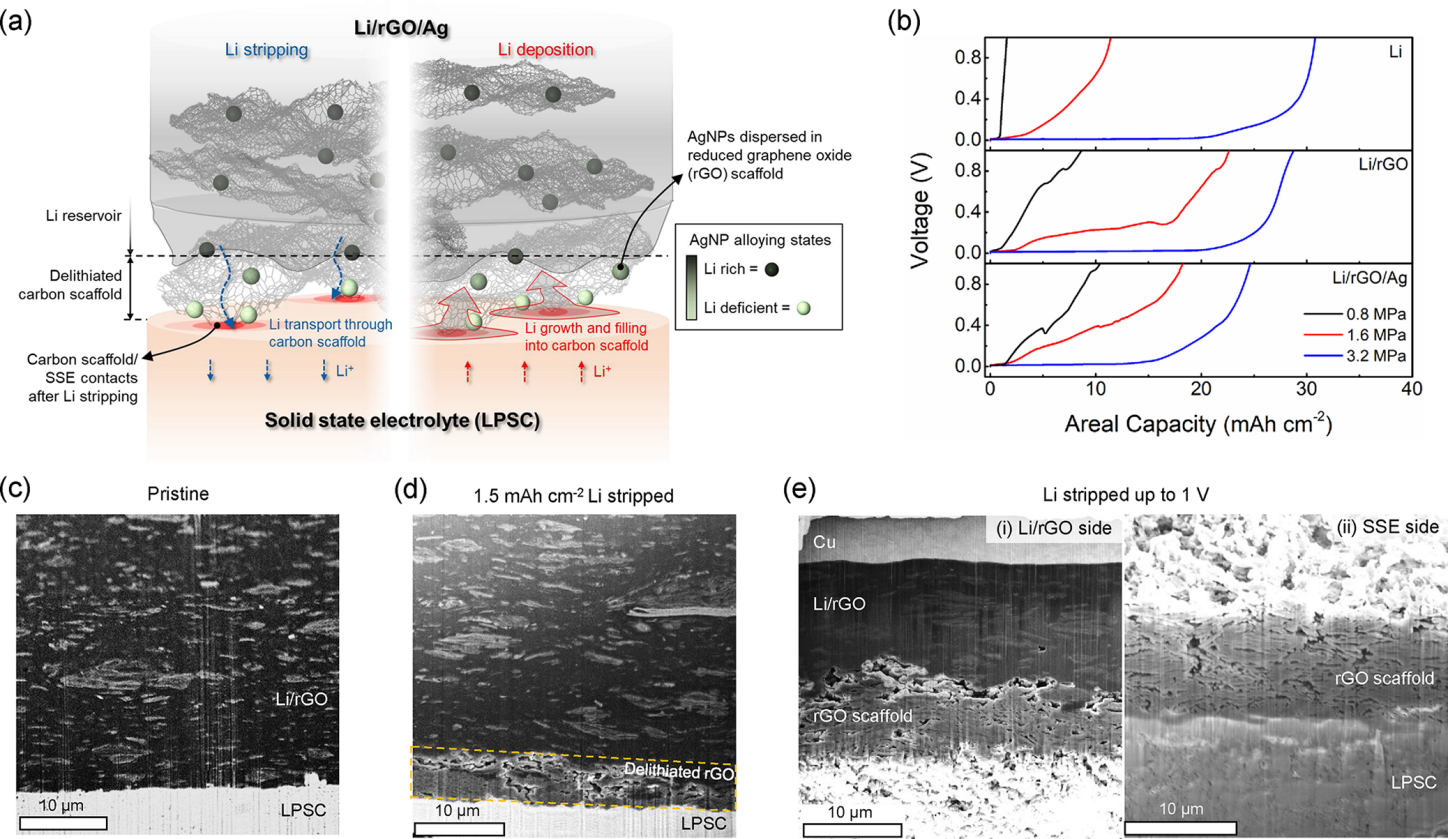
(a) Schematic image of the interface between a Li/rGO/Ag composite anode and a solid-state electrolyte (SSE) during Li stripping and deposition. (b) Voltage profiles as a function of Li stripping capacity using pure Li (top panel), Li/rGO (middle panel), and Li/rGO/Ag (bottom panel) electrodes in half cells with Li_6_PS_5_Cl SSE. The cells featured stack pressures of 0.8, 1.6, or 3.2 MPa and were unidirectionally stripped using a current density of 0.25 mA cm^–2^ at 25 °C. (c–e) Ex situ cryo-FIB-SEM images of the Li/rGO-SSE interface (c) in the initial pristine state, (d) after 1.5 mAh cm^–2^ of Li was stripped, and (e) after exhaustive Li stripping up to a cutoff of 1 V. The samples featured stack pressure of 1.6 MPa and current density of 0.25 mA cm^–2^. After extraction from the cell housing, the cell in (e) spontaneously split into two parts as shown in Figure S4b, with each side imaged separately and shown in (e).

Here, we investigate the electrochemical behavior and structural evolution of Li/C/Ag composite electrodes in SSBs at relatively low stack pressure. The composite electrodes were formed by combining reduced graphene oxide (rGO) with Ag NPs, and then filling with molten Li. During Li stripping from these electrodes, the rGO material accumulates at the SSE interface (Figure 1a), and the Li-depleted carbon scaffold sustains interfacial contact with the SSE and allows for continual Li delivery to the interface. This effect enables greater stripping capacity than pure Li at low stack pressure (1.6 MPa). During redeposition of Li, the distributed Ag NPs act as nucleation centers to induce Li to fill the carbon scaffold (Figure 1a), thereby enabling reversible Li cycling at low stack pressure. Without the particulate alloy inclusion, Li deposition occurs on the surface of the carbon scaffold, which leads to rapid short circuiting. The Ag NPs form a solid solution with Li and thus remain uniformly distributed during Li removal, which promotes homogeneous Li growth throughout the scaffold. Through comparison with other metal NPs, including Au, Si, and Sn, the effect of the nature of alloying on Li cycling behavior is further investigated. Li/rGO/Ag composite anodes were combined with composite sulfur (S) cathodes in full-cell SSBs and cycled at a stack pressure of 4.9 MPa, exhibiting 100 cycles with 79% capacity retention. These findings provide insight into how both the carbon and alloy components synergistically act to reduce the need for high stack pressure during Li plating and stripping, providing guidance toward engineering strategies to improve SSBs.

## Results and Discussion

Li–C composite anodes with and without Ag NPs (hereafter, denoted as Li/rGO and Li/rGO/Ag, respectively) were fabricated via thermal reduction of GO and GO/Ag films in Ar atmosphere, followed by molten Li infiltration (see experimental procedures in the Supporting Information).^35^ After the thermal treatment, expanded pores were observed in both films, which facilitate molten Li infiltration (Figure S1a,b). For electrodes containing Ag, Ag NPs were dispersed in the initial GO solution with a mass ratio of 1:9 (Ag NP:GO) prior to GO film formation. X-ray photoelectron spectroscopy (XPS) spectra showed that the reduced GO and GO/Ag films feature graphitized sp^2^ carbon with minor fractions of sp^3^ carbon bonds and oxygen functional groups (Figure S2a,b). After Li infiltration, the composite electrodes contained 9.9 (±0.7) wt % of rGO. The mass fraction of Ag NPs in the Li/rGO/Ag was ∼1.3 wt %. The composites had a density of 0.53 g cm^–3^, which is similar to pure Li.

Half cells were fabricated with three types of working electrodes (pure Li, Li/rGO, and Li/rGO/Ag) and LPSC electrolyte to analyze Li stripping behavior. Pure Li foil was used as the counter electrode; recent reports with three-electrode cells under similar conditions demonstrated that Li counter electrodes exhibit negligible potential changes during deposition under the conditions used.^16,36^ Our cells were assembled with stack pressures of 0.8, 1.6, or 3.2 MPa and unidirectionally stripped using a current density of 0.25 mA cm^–2^ until polarization. As shown in Figure 1b, the cells showed strikingly different voltage polarization behavior depending on the electrode type and the stack pressure. The mass loading of the electrodes, the stripped capacity, and the stripped Li fraction from each test are in Table S1. All cells with 3.2 MPa stack pressure typically showed flat voltage profiles (<50 mV) followed by rapid polarization after significant areal capacity had been stripped. This suggests retained contact at the stripped interface during this process. The Li, Li/rGO, and Li/rGO/Ag electrodes provided 30.8, 28.8, and 24.6 mAh cm^–2^ of Li stripping capacity up to the cutoff voltage (1 V), corresponding to 78.4%, 83.3%, and 78.3% of the available capacities (Table S1). For lower stack pressures (0.8 and 1.6 MPa in Figure 1b), the total stripped capacities were lower, and in the absence of stack pressure (0 MPa), the stripped capacity was relatively low (<0.8 mAh cm^–2^) and similar for the various electrodes (Figure S3a). This trend suggests an increased extent of contact loss and transport limitations caused by insufficient stack pressures.

Notably, the composite electrodes displayed much higher capacities at 0.8 and 1.6 MPa stack pressure (Figure 1b). For instance, the Li/rGO and Li/rGO/Ag electrodes exhibited 1.8–1.9 times higher Li capacity utilization than the pure Li at 1.6 MPa stack pressure, and 6.3–8.4 times higher at 0.8 MPa stack pressure (Table S1). Similar behaviors were observed at an increased current density of 0.5 mA cm^–2^ (Figure S3b). Differential voltage curves (d*V* d*Q*^–1^, see Figure S3c,d) of the Li stripping profiles in Figure 1b and Figure S3b show that the polarization occurs later in the stripping process in the composite electrodes compared to pure Li. These findings demonstrate that the composite electrodes prevent polarization more effectively than the pure Li at lower stack pressures.

To investigate the evolution of interfacial morphology in the composite electrodes, cross-sectional imaging of the Li/rGO composite electrode-SSE interface at different stages of stripping was carried out with scanning electron microscopy (SEM) combined with cryogenic focused ion beam (cryo-FIB) milling at −140 °C. Cryo-FIB milling can preserve the Li morphology by minimizing sample damage from the Ga^+^ beam.^22,37^ Each half cell was assembled with 1.6 MPa of stack pressure and stripped using a current density of 0.25 mA cm^–2^. Figure 1c shows the pristine Li/rGO-SSE interface prior to stripping, in which lighter-contrast carbon flakes are uniformly distributed throughout the Li matrix (Figure S4a shows energy dispersive spectroscopy (EDS) data). After stripping 1.5 mAh cm^–2^ (Figure 1d), porous carbon devoid of Li is accumulated at the SSE interface as a layer 5–6 μm in thickness. When the Li was fully stripped up to 1 V cutoff voltage (Figure 1e), the extracted cell split at the delithiated rGO layer during disassembly, as shown in Figure S4b. The significant stripping of Li resulted in accumulation of a much thicker layer of the delithiated, porous rGO scaffold at the interface (Figure 1e and S4c). XPS analysis showed that the rGO scaffold was primarily composed of the graphitized carbon along with minor sp^3^ C and oxygen derivatives, similar to the rGO film in the pristine state (Figure S2a,c).

**Figure 2:**
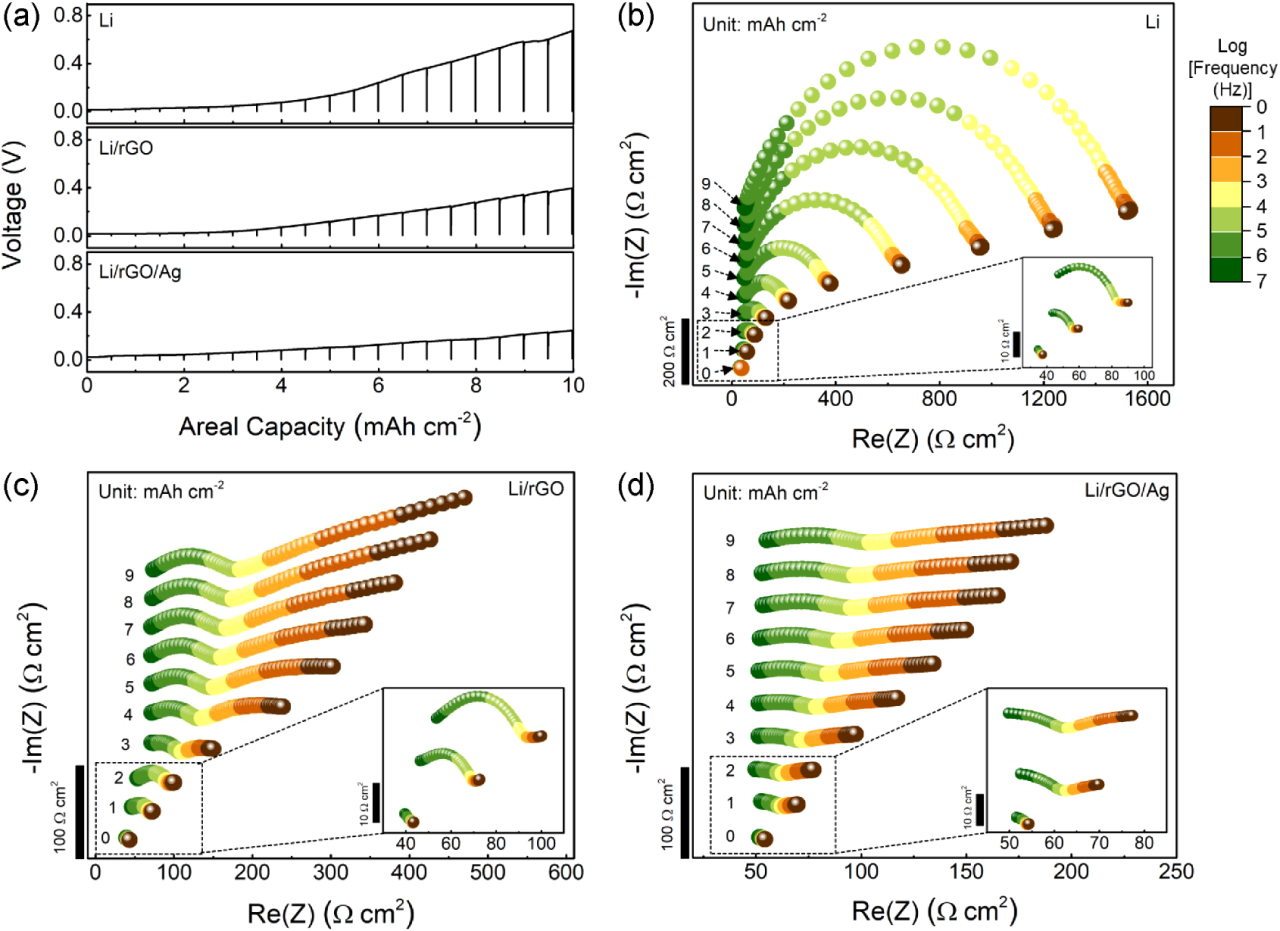
In situ EIS analysis during Li stripping from various electrodes. (a) Li stripping voltage responses for the Li (top panel), Li/rGO (middle panel), and Li/rGO/Ag (bottom panel) working electrodes in half cells. Potentiostatic impedance spectra were collected periodically at intervals of 0.5 mAh cm^–2^; vertical lines in the voltage profiles denote the EIS measurements. The cells were assembled with 1.6 MPa stack pressure and tested using 0.25 mA cm^–2^ current density. (b–d) Impedance spectral evolution of (b) pure Li, (c) Li/rGO, and (d) Li/rGO/Ag electrodes between 0 and 9 mAh cm^–2^ stripped, with the insets showing magnified views of the first three spectra. Tests were performed at 25 °C.

Electrochemical impedance spectroscopy (EIS) was performed to further understand interfacial evolution of the composite electrode. Half cells using Li, Li/rGO, and Li/rGO/Ag working electrodes were operated with 1.6 MPa of stack pressure. EIS was carried out during stripping from the working electrode at periodic intervals of 0.5 mAh cm^–2^ using a current density of 0.25 mA cm^–2^ (Figure 2a). During stripping of 10 mAh cm^–2^ areal capacity, the pure Li, Li/rGO, and Li/rGO/Ag electrodes in the half cells showed monotonic voltage increases up to 0.67, 0.4, and 0.25 V, respectively (Figure 2a). The obtained Nyquist plots are shown in Figure 2b–d. Since void evolution at the interface can cause 3D current distributions that make conventional linear equivalent circuit analysis unreliable,^38,39^ the obtained Nyquist plots were only analyzed qualitatively.

The impedance evolution of the pure Li electrode was quite different from the composite electrodes during this process. As shown in Figure 2b–d, the Nyquist plots from all cells initially featured a portion of a semicircle with intercepts at relatively low impedance (<60 Ω cm^2^). During stripping, the pure Li electrode exhibited a large increase in the width of this semicircle from a few Ω cm^2^ to ∼1500 Ω cm^2^ after 9 mAh cm^–2^ had been stripped (Figure 2b). The apex frequency of the semicircle shifted from ∼1 MHz to ∼10 kHz as the diameter grew (Figure S5a). This semicircle growth can be attributed to interfacial contact loss at the Li working electrode.^4,40^ There are other interfacial processes that could contribute, such as charge transfer or solid-electrolyte interphase resistances,^41^ but recent reports suggest that the apex frequency shift is likely associated with the evolution of 3D voids and corresponding interfacial Li-SSE contact changes.^38,39^ The growth of the semicircles from both composite electrodes was much less, with final widths of 180 and 100 Ω cm^2^ at 9 mAh cm^–2^ for the Li/rGO and the Li/rGO/Ag electrodes, respectively. The apex frequencies of both impedance curves were shifted from ∼1 MHz to ∼100 kHz (see also Figure S5b,c). In contrast to the pure Li, the composite electrodes also featured extension of low-frequency tails from 10 kHz to the lower frequency limit during stripping. At early stages of the stripping process (between 2 and 4 mAh cm^–2^), both showed second semicircular features at ∼10 kHz (Figure S5d,e), with inflection points of ∼100 Hz (red arrows in Figure S5b,c). However, these features straightened into low-frequency tails after further stripping (Figures 2c,d and S5d,e).

The accumulation of the delithiated carbon scaffold at the SSE interface during stripping is likely responsible for the different impedance evolution of the composite electrodes. Accumulation of the carbon at the interface instead of voids could maintain homogeneous interfacial contact with the SSE, suppressing the interfacial resistance increase, as shown in Figure 2c,d. Notably, the overall interfacial resistances during stripping were slightly lower at the Li/rGO/Ag electrode compared to the Li/rGO electrode, which might be associated with a stabilizing role of Ag at the carbon scaffold-SSE interface.^33^ The growth of the extended tails at frequencies below 10 kHz is likely associated with long-range transport of Li through the growing delithiated rGO layer from the Li metal in the composite.^42,43^ Hence, this phenomenon can be regarded as a finite-length diffusion layer with a transmissive boundary (the rGO-SSE interface), which is presented as a slanted straight line at low frequency (Warburg impedance).^44−46^ The impedance spectra of the composite electrodes are also more vertically compressed compared to the pure Li (Figure 2b–d), since the evolution of additional boundaries via the rGO (or rGO/Ag) scaffold formation can require multiple Li transfer processes^46^ or bring about microstructural irregularities at each boundary.^47^ Replacing the interfacial voids (dielectric) with the conductive scaffold (rGO and Ag NPs) makes the interface less capacitive, which is responsible for the lower scale of imaginary impedance in the composite electrodes (Figure S5a–c). During stripping, then, the carbon scaffold becomes progressively emptied near the SSE interface, but it retains contact with the interface and still facilitates Li transport via surface diffusion on the high-surface-area carbon. This effect largely bypasses the voiding behavior of pure Li anodes.

**Figure 3:**
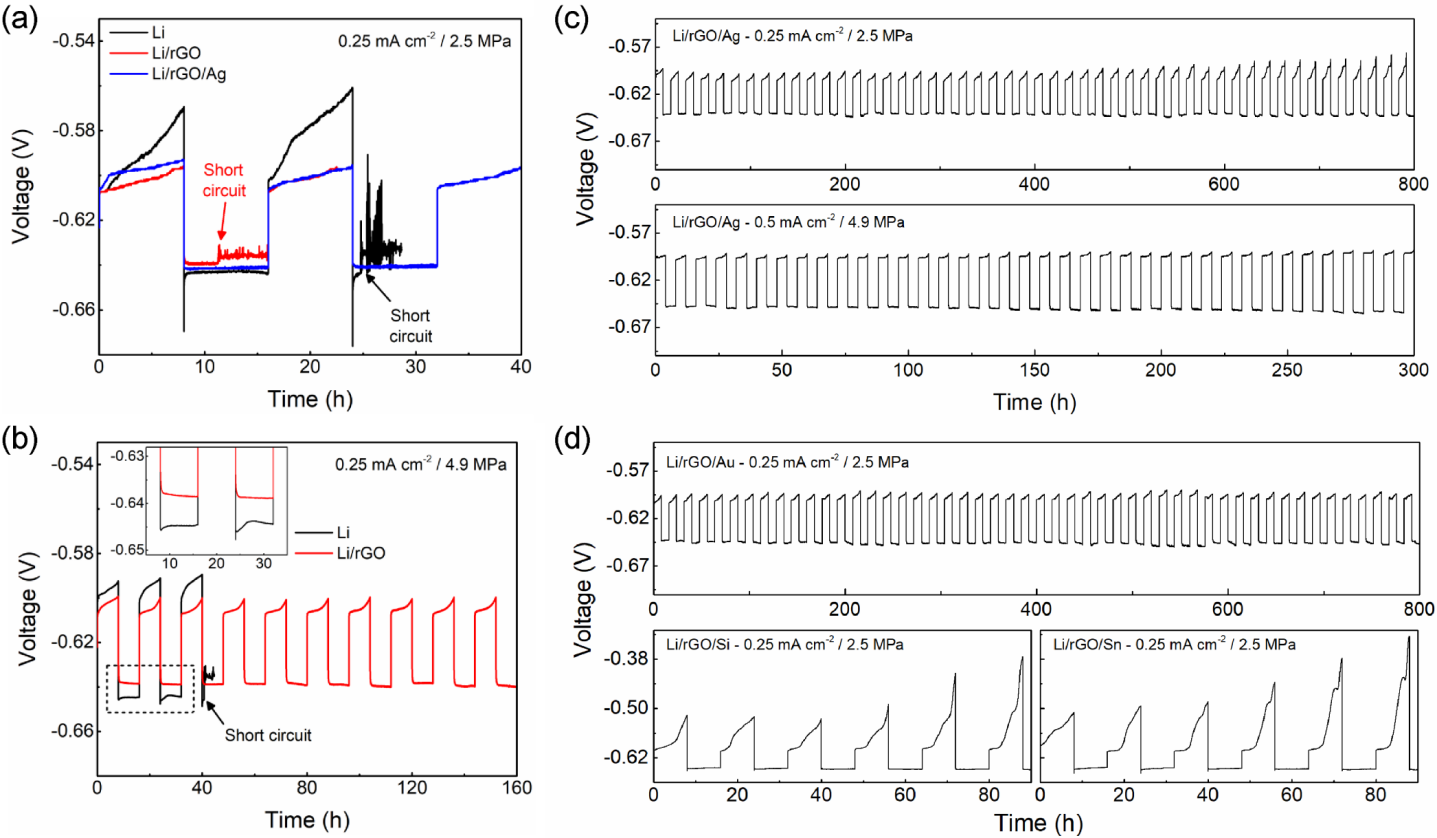
Galvanostatic cycling tests of the pure Li and Li composite working electrodes with LiIn counter electrodes, which exhibit a potential of 0.62 V vs. Li/Li^+^. (a) Cycling tests of the pure Li, Li/rGO, and Li/rGO/Ag electrodes at 2.5 MPa stack pressure. The difference in initial stripping voltage between Li/rGO and Li/rGO/Ag is likely due to statistical variation among electrodes. (b) Cycling tests of the pure Li and Li/rGO electrodes at 4.9 MPa stack pressure. The inset graph in (b) shows enlarged voltage profiles from the black rectangular region. A current density of 0.25 mA cm^–2^ was used in (a) and (b). (c) Cycling tests of the Li/rGO/Ag electrodes at 2.5 MPa stack pressure using 0.25 mA cm^–2^ current density (top) and at 4.9 MPa stack pressure using 0.5 mA cm^–2^ current density (bottom). The first two cycles in the top panel of (c) are also shown in (a) for a comparison with the pure Li and Li/rGO electrodes. (d) Galvanostatic cycling tests of the Li composite electrode using different metal NPs; Au (top), Si (bottom left), and Sn (bottom right). The cells were tested under identical conditions as the test shown in (a). All tests were conducted at 25 °C.

To understand the influence of the composite structure on Li cycling, galvanostatic cycling tests were carried out using the pure Li, Li/rGO, and Li/rGO/Ag electrodes in half cells. The cells were constructed using the Li_1_In_3_ alloy (LiIn) counter electrode. The wide stoichiometric range and constant redox potential (0.62 V vs Li/Li^+^) enables this alloy to function as a reliable counter electrode over many cycles in SSB systems.^41,48^ A stack pressure of 2.5 MPa was applied to the cells, and the cells were cycled using a current density of 0.25 mA cm^–2^ and areal capacity per cycle of 2 mAh cm^–2^. Figure 3a shows typical voltage profiles of the first and second cycles. All the cells began with Li stripping from the working electrode and displayed similar initial overpotentials (∼13 mV). With additional stripping, however, the pure Li electrode displayed a steeper voltage change than the Li/rGO and Li/rGO/Ag electrodes, which is consistent with the result shown in Figure 1b. The Li and Li/rGO electrodes featured short circuits within two cycles. The Li/rGO/Ag electrode, on the other hand, successfully cycled over 50 times (800 h) at this stack pressure (top panel of Figure 3c). When tested at the lower stack pressure of 1.6 MPa, the Li/rGO/Ag electrode continued to display steady cycling without short circuiting but became increasingly polarized upon repeated cycling (Figure S6a). This indicates that at least a certain level of stack pressure, which in this case is greater than 1.6 MPa, is required to maintain cycling reversibility. Li/rGO/Ag electrodes also showed stable cycling over 30 cycles with twice the current density (0.5 mA cm^–2^) at the higher stack pressure of 4.9 MPa (bottom panel of Figure 3c). As shown in Figure 3b, the cycling behavior of the Li and Li/rGO electrodes was examined again at a current density of 0.25 mA cm^–2^ but with a doubled stack pressure of 4.9 MPa. The pure Li electrode featured less sloping overpotentials than that tested at 2.5 MPa but still short circuited in the third cycle. The Li electrode exhibited stable cycling with the higher stack pressure of 8.1 MPa (Figure S6b). The Li/rGO electrode showed stable cycling without short circuiting at stack pressures of 4.9 MPa and above (Figures 3b and S6c). These results imply that the minimum stack pressure that allows for stable cycling is the lowest for the Li/rGO/Ag electrode (2.5 MPa) and increases in the series Li/rGO/Ag < Li/rGO < Li.

A Li/Ag electrode without the carbon filler was also examined to understand the effect of the carbon scaffold (Figure S6d). The Li/Ag electrode was created by mixing the same amount of Ag NPs into the molten Li as for the Li/rGO/Ag electrode (∼1.3 wt %), and the same conditions as in Figure 3a (0.25 mA cm^–2^ and 2.5 MPa) were employed for the testing. The voltage profile exhibited a sloping overpotential in the first Li stripping step, which was similar to the result from pure Li case (Figure 3a), followed by a short circuit during the deposition step. This result demonstrates that stable cycling of a Li/rGO/Ag electrode at the lowest stack pressure (2.5 MPa) is only feasible through the synergistic effect of both carbon layer at the interface and the dispersed Ag NPs. It is also noteworthy that the Li/rGO and Li/rGO/Ag electrodes displayed flat voltage curves at the very beginning of plating, in contrast to the pure Li (Figure 3a,b) and the Li/Ag electrodes (Figure S6d), which showed a nucleation overpotential. This behavior indicates that the carbon interlayers formed in situ during stripping aid Li deposition in subsequent plating steps.

The impact of the nature of the alloy particles on Li cycling was further investigated by employing different NPs (Au, Si, and Sn) hosted within rGO-based composite electrodes prepared identically to the Li/rGO/Ag (Figure 3d). The size of the NPs was typically less than 150 nm (Table S2). The mass ratio of the rGO to the NPs was 9:1 for all samples for consistency (Table S2 tabulates atomic fractions). The Li/rGO/M half cells were assembled using LiIn counter electrodes and electrochemically cycled at a stack pressure of 2.5 MPa, 0.25 mA cm^–2^ current density, and 2 mAh cm^–2^ areal capacity. The results in Figure 3d show that only the Li/rGO/Au electrode exhibited stable cycling over 50 cycles without noticeable polarization, while the Si and Sn composite electrodes exhibited steep voltage increases during Li stripping which became exacerbated over the first six cycles. Figure S7 compares the initial voltage profile of these composite electrodes. Compared to the Ag and Au composite electrodes, the Si and Sn composite electrodes displayed greater increases of the stripping overpotential followed by sharp spikes in the subsequent plating steps. This suggests that only Ag and Au NPs can disperse Li so that it is transported effectively through the carbon layer during the stripping step.

**Figure 4:**
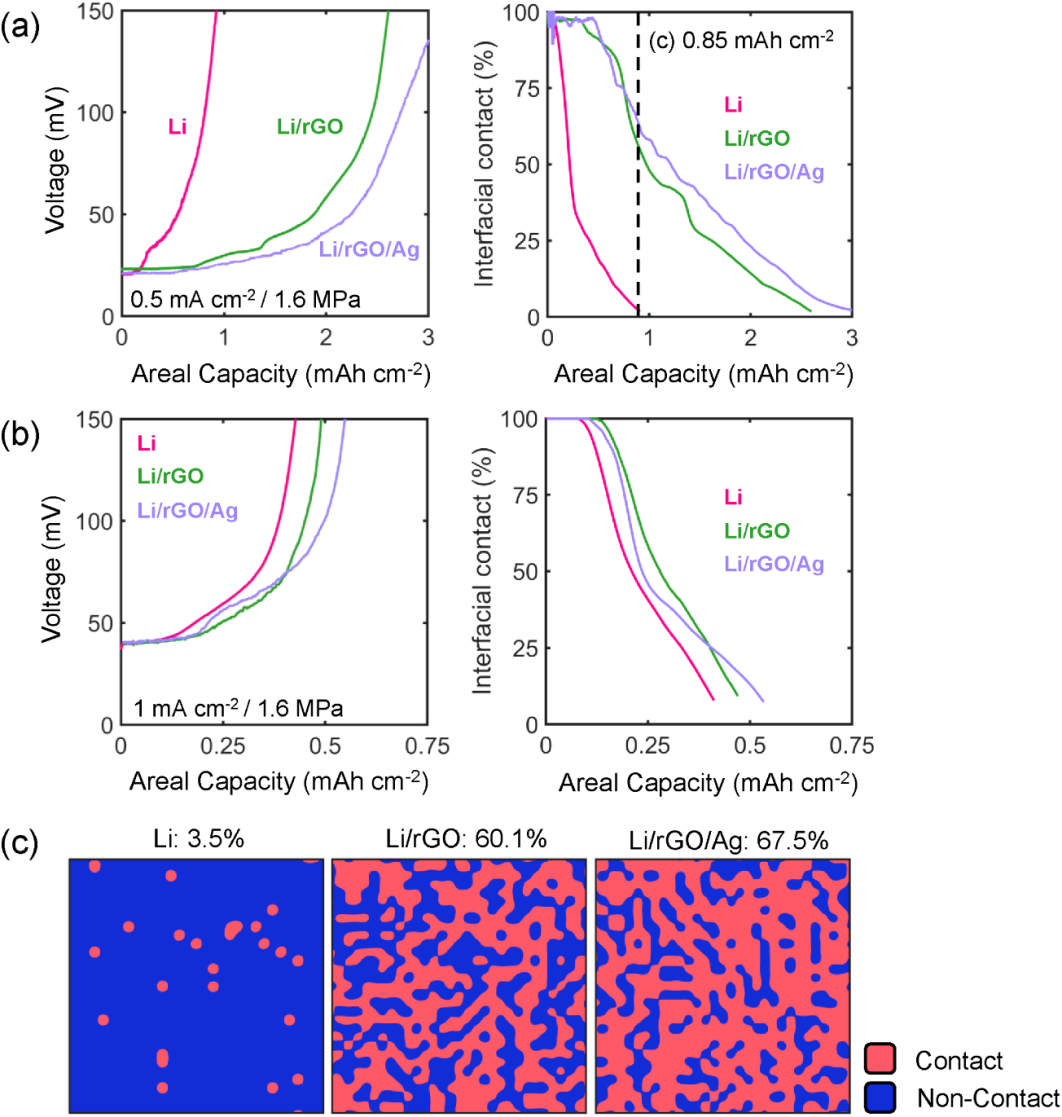
(a,b) Experimental voltage curves during galvanostatic stripping (left panels) and predicted interfacial contact evolution (right panels) of the pristine Li, Li/rGO, and Li/rGO/Ag interfaces for (a) 0.5 mA cm^–2^ and (b) 1 mA cm^–2^ current densities at 1.6 MPa stack pressure. (c) Contact maps at 0.85 mAh cm^–2^ of stripped capacity for the operating conditions in (a). The dimensions of the contact maps are 15 × 15 μm^2^, and the values above each map are the percentage of retained contact area.

To understand how the presence of the scaffold influences interfacial contact during stripping, we developed an electrochemical model that predicts contact loss behavior based on experimentally obtained cell voltage profiles. The formation of voids without Li transport pathways results in a higher kinetic overpotential at the interface and a higher ionic transport resistance in the vicinity of voids due to current constriction. In the composite electrode case, we consider that contact of the delithiated carbon scaffold at the SSE can still allow for Li transport to the interface, but likely with different transport characteristics than pure Li. The mathematical formulation used in the model to develop the correlation between the cell voltage and the contact evolution during stripping is described in the Supporting Information. As shown in the experimental electrochemical stripping curves (Figure 4a, left panel), a nonlinear increase in the voltage profile occurs at a much earlier capacity for Li when compared to Li/rGO and Li/rGO/Ag at a current density of 0.5 mA cm^–2^ and stack pressure of 1.6 MPa. The contact evolution trends from the model (Figure 4a, right panel) reveal that a rapid decrease of interfacial contact occurs for Li during this process, resulting in almost complete loss of contact (3.5% contact area retention) at 0.85 mAh cm^–2^ of stripped capacity. At the same stripped capacity, substantially larger contact fractions (60–70%) are obtained for Li/rGO and Li/rGO/Ag electrodes, demonstrating their ability to facilitate improved contact retention. For a higher stripping current density of 1 mA cm^–2^ with 1.6 MPa stack pressure (Figure 4b, left panel), the contact profiles of all three types of electrode decay at a faster rate, with only minor differences between them (Figure 4b, right panel). At these higher currents, this trend suggests a reaction-dominated regime and the need for a higher pressure to improve the interfacial contact in all cases. A similar comparison between Li, Li/rGO and Li/rGO/Ag at the intermediate current density of 0.75 mA cm^–2^ and 1.6 MPa stack pressure is shown in Figure S8, again showing improved contact retention for the composite electrodes.

For the stripping condition shown in Figure 4a, 2D maps of interfacial contact at a stripping capacity of 0.85 mAh cm^–2^ are shown in Figure 4a for Li, Li/rGO and Li/rGO/Ag, illustrating differences in the contact distribution. The details regarding the construction of the contact distributions from the voltage response are given in the SI. The distribution of contact and noncontact points and the associated reaction distribution at the interface dictate the kinetic overpotential (see Figure S9) and in turn the overall cell voltage. Importantly, the rapid contact loss of pure Li results in the formation of isolated contact points (Figure 4c), while Li/rGO and Li/rGO/Ag exhibit more connectivity between the contact regions, which is consistent with the delayed increase of interfacial impedance shown in Figure 2. The isolated contact points for the Li electrode can serve as local hot spots for filament growth during a subsequent plating step and lead to accelerated cell failure. Thus, the improved contact retention of Li/rGO and Li/rGO/Ag during stripping can enhance cycling performance at low stack pressures.

**Figure 5:**
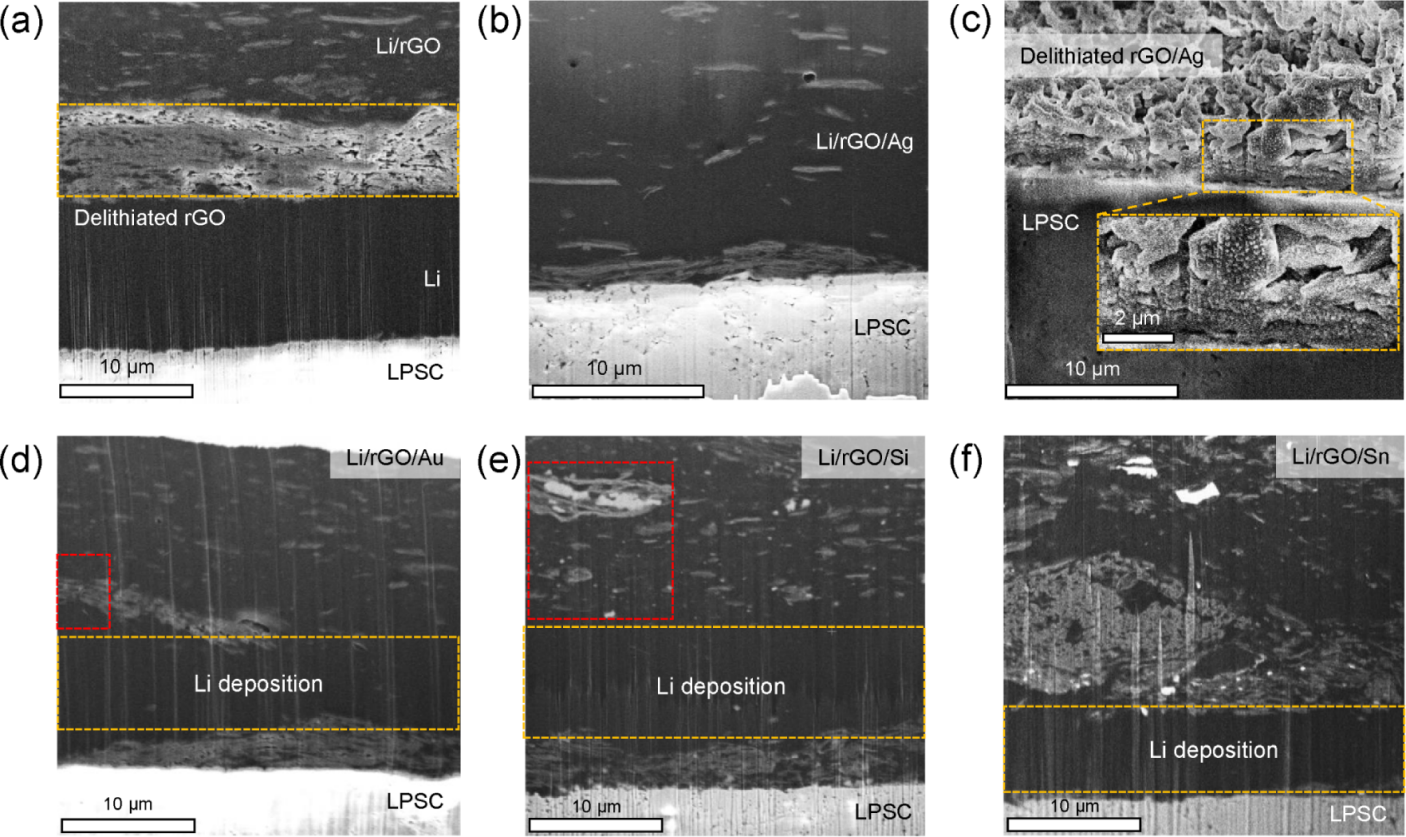
(a–c) Ex situ cryo-FIB SEM images of the (a) Li/rGO-SSE and (b) Li/rGO/Ag-SSE interfaces after stripping 4 mAh cm^–2^ followed by plating 2 mAh cm^–2^ of Li, and (c) the Li/rGO/Ag-SSE interface after stripping to 1 V upper cutoff in half cells. The cells were assembled and tested at 1.6 MPa stack pressure with 0.25 mA cm^–2^ current density at 25 °C. The cell in which the Li/rGO/Ag cell was fully stripped of Li was easily split at the region of the rGO scaffold, and the rGO/Ag scaffold-SSE interface was used for the imaging. (d–f) Ex situ cryo-FIB SEM images of the (d) Li/rGO/Au-SSE, (e) Li/rGO/Si-SSE, and (f) Li/rGO/Sn–SSE interfaces after stripping 4 mAh cm^–2^ and replating 2 mAh cm^–2^ of Li. EDS line scans and maps from the red boxes in (d) and (e) are shown in Figure S14.

We further experimentally investigated evolution of these composites during charge/discharge cycling. Li/rGO and Li/rGO/Ag half cells were constructed and cycled under 1.6 MPa of stack pressure using a current density of 0.25 mA cm^–2^. An areal capacity of 4 mAh cm^–2^ was first stripped from the working electrodes, followed by replating 2 mAh cm^–2^ of Li. Figure 5a,b shows cross-sectional cryo-FIB SEM images of the Li/rGO and Li/rGO/Ag electrodes. Plating on the stripped Li/rGO electrode resulted in Li deposition directly at the delithiated rGO/SSE interface, with a dense ∼10 μm Li layer visible between the delithiated rGO and SSE (Figure 5a). The rGO layer is clearly still porous, as Li primarily grew on the surface of the layer and not inside the pores; this indicates that Li does not preferentially nucleate in or wet the rGO.^32^ In contrast, after Li deposition on the Li/rGO/Ag electrode, the rGO/Ag scaffold was completely filled with the deposited Li, with no obvious rGO layer remaining. The incorporation of the Ag NPs can therefore enhance the nucleation and growth behavior of Li within the carbon scaffold, causing the deposited Li to fill the scaffold instead of depositing on the surface. Ag is known to favor Li nucleation,^27,49^ and in this case, it appears to spatially control the Li growth behavior. This behavior could be beneficial since incorporation of Li within the carbon scaffold enables uniform growth without substantial applied stack pressure,^24,32^ which is likely the reason for the superior cycling of the Li/rGO/Ag electrode at the lowest stack pressure (Figure 3a,c).

**Figure 6:**
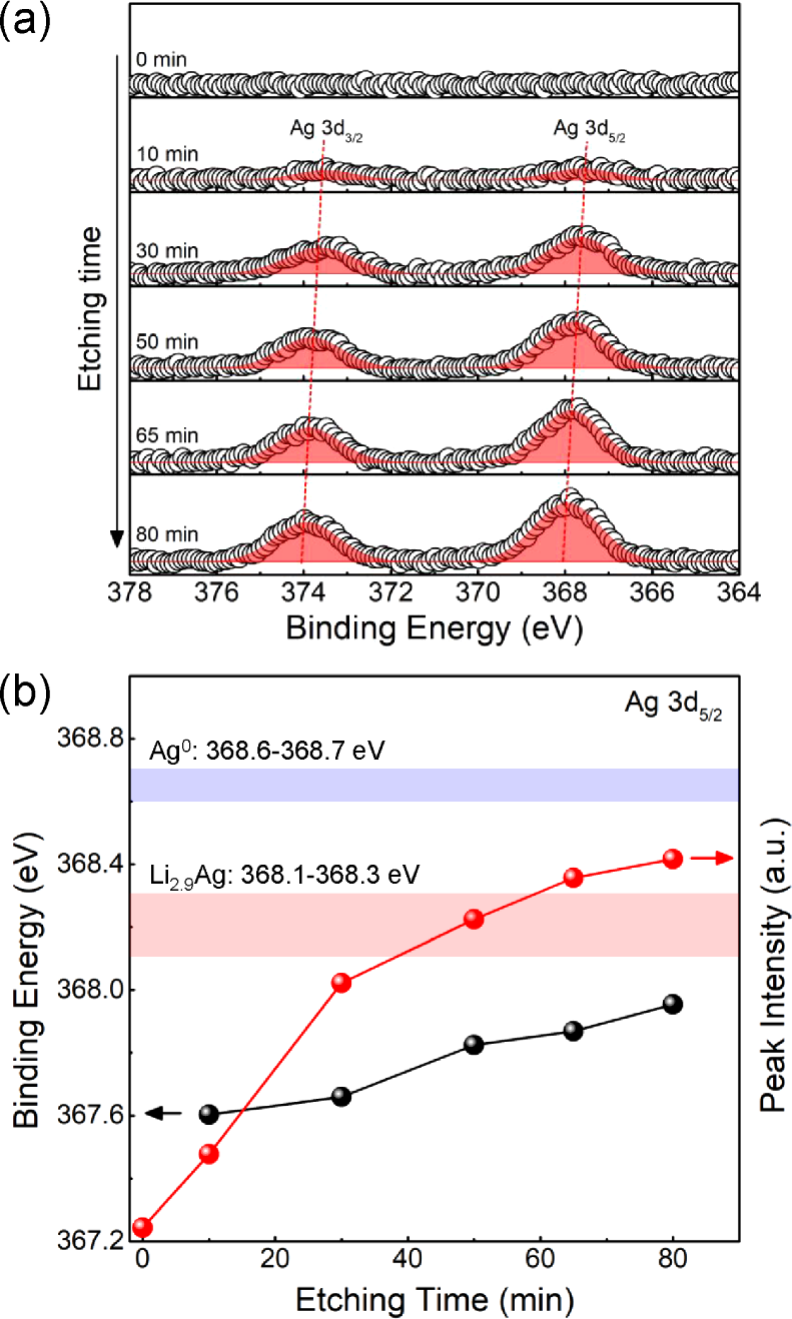
(a,b) XPS analysis of the delithiated rGO/Ag scaffold layer shown in Figure 5c. (a) Depth-resolved Ag 3d spectra of the rGO/Ag scaffold. Two peaks, Ag 3d_3/2_ and Ag 3d_5/2_, were observed with a binding energy difference of 6 eV. (b) Binding energy and intensity of the Ag 3d_5/2_ peaks as a function of etching time (i.e., depth of etching). Increased etching time indicates a closer position to the SSE interface. The binding energy bands for Ag^0^ (blue) and Li_2.9_Ag (red) are included for reference.

Further investigation of a Li/rGO/Ag electrode was carried out with cryo-FIB SEM (Figure 5c) and XPS (Figure 6) after stripping Li from the composite in a half cell to an upper voltage cutoff of 1 V. Figure 5c shows a cryo-FIB SEM image of the cross section near the LPSC interface. The delithiated rGO/Ag scaffold is similar to the delithiated rGO scaffold in Figures 1e and S4c. However, Ag particles with size of ∼100 nm are visibly dispersed on the rGO scaffold (see enlarged image in Figure 5c and EDS line scan in Figure S10). Thus, Ag NPs remain throughout the scaffold after Li removal. Depth-resolved XPS was used to track the spatial distribution of the Ag particles within the scaffold. As shown in Figure 6a, the two peaks of the Ag 3d doublet (Ag 3d_3/2_ and Ag 3d_5/2_) were observed with binding energies of ∼374 and ∼368 eV. This Figure shows the spectra after different plasma etching times to probe through the depth of the sample from the back of the delithiated rGO scaffold toward the LPSC interface. Figure 6b displays the binding energy and intensity changes of the Ag 3d_5/2_ peak with etching time. The peak intensity increased with depth, and the peak position was also slightly shifted toward higher binding energies. The increase of Ag 3d peak intensities deeper into the sample demonstrates that the Li removal from the Li/rGO/Ag electrode caused the Ag particles to redistribute closer to the SSE interface. Before the Li infiltration into the rGO/Ag film, the Ag particles were evenly distributed throughout the scaffold, as shown by the control XPS data in Figure S11b.

To understand the chemical state of the Ag particles, binding energy positions of pristine Ag (Ag^0^) and electrochemically lithiated Ag (Li_2.9_Ag) are shown as the blue and red reference bands in Figure 6b. The Ag^0^ binding energy was obtained from a pure Ag foil and a rGO/Ag film before the Li infiltration, which consistently exhibited Ag 3d_5/2_ peaks at 368.6–368.7 eV (Figure S11). The Li_2.9_Ag binding energy was obtained from an Ag foil lithiated to a cutoff of 0 V (Figures S12 and S13a, and Supporting Note 1). Electrochemical (Figure S12a) and XRD characterization (Figure S13b) showed that this sample contained alloy phases of Li_9_Ag_4_ and Li_3_Ag. As shown in Figure S13a, the Ag 3d_5/2_ peak of the Li_2.9_Ag foil exhibited lower binding energies (368.1–368.3 eV) than that for Ag^0^. The Ag particles in the Li/rGO/Ag electrode were originally chemically lithiated through contact with molten Li, with low atomic fraction (0.08 at. %) of Ag present (Table S2), meaning the Li–Ag alloys are initially in a Li-dominant solid solution phase.^28,29^ The binding energies after Li metal removal from the scaffold were still lower than the Li_2.9_Ag band in Figure 6b, suggesting that the Ag particles were more Li-rich than Li_2.9_Ag even after Li metal removal from the scaffold. Ag can become highly lithiated due to spontaneous alloying during Li deposition, as shown in recent reports^27^ and demonstrated in Figure S12. The extensive chemical alloying of Ag with Li could provide a favorable environment for continued Li deposition within the rGO/Ag scaffold, as directed by Ag.

To understand why the presence of Ag enabled better cycling than Sn or Si NPs, cryo-FIB imaging was carried out on composites with different alloy particles. Figures 5d–f show ex situ cryo-FIB SEM images of the Au, Si, and Sn composite electrodes after stripping 4 mAh cm^–2^ and replating 2 mAh cm^–2^ of Li. Similar to the Li plating on the rGO/Ag scaffold (Figure 5b), the deposited Li mostly filled the various rGO/metal particle scaffolds, in contrast to the growth of Li on the surface of porous scaffolds without the NPs. For the Si and Sn composite electrodes, however, localized alloy phases (the brightest regions) were scattered throughout the cross sections in Figure 5e, f. Such nonuniform distributions were not observed within the Au composite electrode (Figure 5d). The Au can completely dissolve into Li to form a solid solution phase due to the relatively small Au concentrations present, similarly to how Ag forms a solid solution with Li.^28,29^

During Li stripping, the Ag and Au dissolved within Li precipitate out of the solution and can redistribute uniformly throughout the carbon scaffold, as demonstrated in Figure 5c for Ag. This behavior enhances the morphological reversibility of Li deposition by providing uniformly distributed NPs within the rGO/M scaffold from which Li can grow in the subsequent deposition step (Figure 5b and d). In contrast, given the high concentration of Li in the composites (Table S2), the Si and Sn will form highly lithiated intermetallic alloys such as Li_15_Si_4_ and Li_22_Sn_5_.^50,51^ Since they are insoluble, these intermetallic alloys are responsible for the nonuniform distribution of the lithiated phases (Figure 5e,f).^28,50^ Although the insoluble alloy particles can provide a lithiophilic environment to aid the Li growth within the rGO/M scaffolds, as shown in Figure 5e,f, these localized particles do not become uniformly redistributed each stripping half-cycle, leading to current concentrations and poor cyclability (Figure 3d). This finding suggests that the morphological reversibility of Li during cycling of the composite electrodes is strongly influenced by the solid solution vs intermetallic nature of the Li alloy phases, since dissolution in a solid solution allows for more uniform spatial redistribution of the alloy component each cycle.

**Figure 7:**
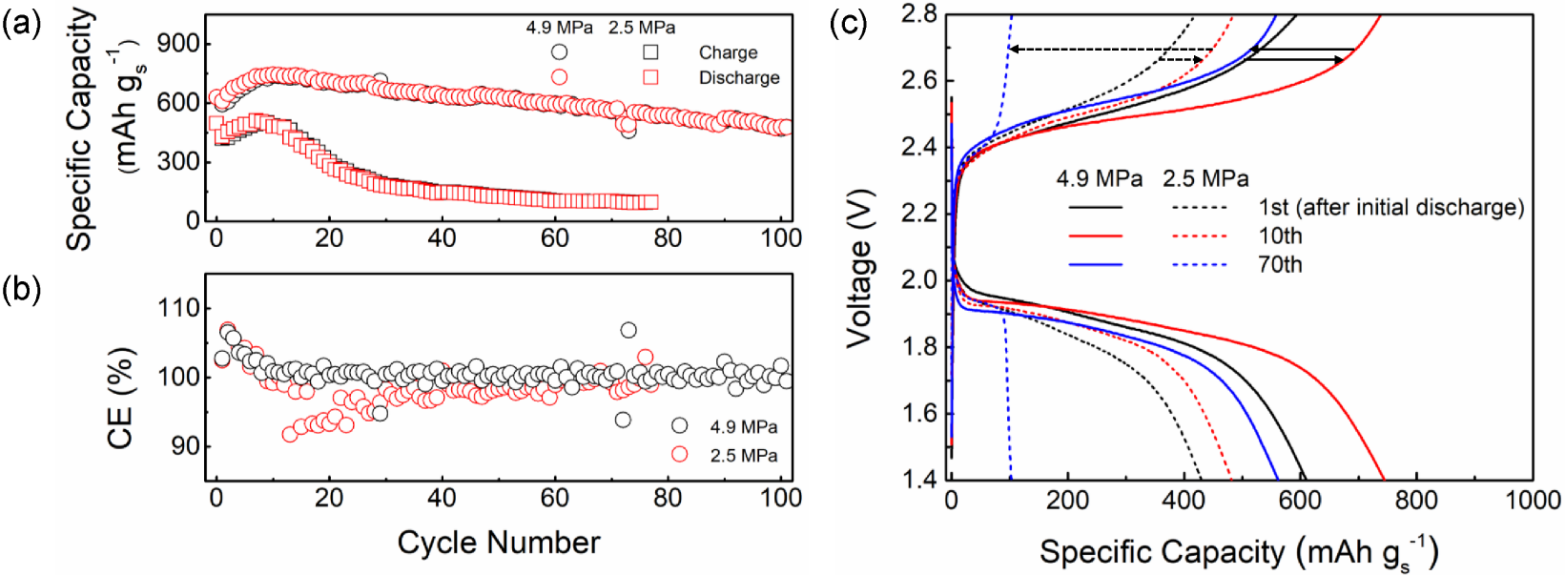
Galvanostatic cycling tests of Li/rGO/Ag || S full cells at different stack pressures and a temperature of 25 °C. The cells were discharged at 0.1 mA cm^–2^ to activate the S cathode, and then they were cycled at 0.25 mA cm^–2^ (0.043 C). The sulfur loadings within the 4.9 and 2.5 MPa cells were 3.5 and 3.4 mg cm^–2^, respectively, corresponding to 5.82 and 5.76 mAh cm^–2^ theoretical areal capacity. Lithium loadings in the anode side were 4.5–4.7 times these capacities (meaning a high N:P ratio). (a) Specific charge and discharge capacities and (b) Coulombic efficiency (CE) of the cells at 4.9 and 2.5 MPa stack pressure. (c) Voltage profiles of the first, 10th, and 70th cycle after the initial discharge step at both stack pressures.

To demonstrate the behavior of the composite electrodes in full cells, we combined Li/rGO/Ag anodes with sulfur-based cathodes and evaluated electrochemical behavior at different stack pressures (Figure 7). Sulfur cathodes were chosen instead of conventional intercalation cathodes such as LiNi_*x*_Co_*y*_Mn_*z*_O_2_ (NMC) since the composite anode contains Li, and it is preferable to strip in the first half-cycle to generate the carbon scaffold. A sulfur-based composite containing multiwalled carbon nanotubes (CNTs) and LPSC was used (mass ratio of 2:1:3 S:CNTs:LPSC, see the Methods and Experimental section).^52,53^ The cells were loaded with ∼3.5 mg cm^–2^ of S and operated with 4.9 or 2.5 MPa stack pressure and 0.25 mA cm^–2^ current density (0.043 C, 1 C = 1672 mA g_S_^–1^). Both cells underwent an initial discharge with 0.1 mA cm^–2^ current density to activate the S cathode. The 4.9 and 2.5 MPa cells delivered initial discharge capacities of 629 mAh g_S_^–1^ (2.19 mAh cm^–2^) and 497 mAh g_S_^–1^ (1.71 mAh cm^–2^), respectively (Figure 7a). The 4.9 MPa cell underwent over 100 cycles without short circuiting, while the 2.5 MPa cell underwent 79 cycles and then short circuited (Figure 7a). The 4.9 MPa cell had a specific discharge capacity of 609 mAh g_S_^–1^ for the first cycle, which increased to 744 mAh g_S_^–1^ after 10 cycles. Almost 79% of the initial capacity (479 mAh g_S_^–1^) was retained after 100 cycles with a high average Coulombic efficiency (CE) of 99.7% over cycles 10–100 (Figure 7b). In contrast, the discharge capacity of the 2.5 MPa cell was 429 mAh g_S_^–1^ at the first cycle and increased to 511 mAh g_S_^–1^ at the seventh cycle. It decreased to less than 25% of the first discharge capacity after 50 cycles (124 mAh g_S_^–1^) with an average CE of 97.7% over cycles 10–79 (Figure 7b). A cell with a pure Li anode at 4.9 MPa stack pressure was tested as a control experiment, which showed short circuiting after 6 cycles (Figure S15).

Figure 7c shows voltage profiles from the first, 10th, and 70th cycles after the initial discharge step. Overpotential variations between the two cells were minor initially, but in the later cycles, the 2.5 MPa cell exhibited steeper voltage changes than the 4.9 MPa cell. Greater internal resistances within the S cathodes could be responsible for the worse cyclability in the 2.5 MPa cell. Given that the 4.9 MPa cell was able to reach 100 cycles with 79% retention, this higher stack pressure is still important for both delivering higher capacity from the S cathode and retaining that capacity over extended cycling. The stack pressures used in this study are substantially lower than those used in previous investigations on S-based cathodes (>1000 mAh g_S_^–1^ with >50 MPa),^53,54^ and more work is needed to understand the influence of stack pressure on connectedness within conversion cathodes.^52^ Our composite anode, on the other hand, behaves well at even lower stack pressures, demonstrating the utility of combining materials with different functions into the composite (Ag and C). To improve full cell performance at low stack pressures, incorporating higher-conductivity materials into the cathode or engineering composite cathode architectures to retain particle contact may be necessary.^52,54,55^

## Conclusions

In this study, we investigated the evolution of the electrode-SSE interface during Li stripping from Li/rGO composite electrodes with and without Ag, and the effects of both the carbon scaffold and the distributed Ag particles on Li cycling at low stack pressures were identified. The rGO scaffold accumulates at the SSE interface during stripping of Li, and this scaffold can beneficially function as bridging material to deliver Li to the SSE while retaining homogeneous contact to the SSE during Li stripping, which significantly enhances the stripping capacity at low stack pressures (<3.2 MPa). The sustained contact at the rGO scaffold-SSE interface was also effective for enhancing subsequent Li deposition, thereby achieving stable cycling at a stack pressure of 4.9 MPa. However, Li tends to grow directly on the surface of the conductive carbon scaffold without Ag present, increasing the risk of cell failure via dendritic deposition. The Li/rGO/Ag electrodes exhibited better cyclability than the Li/rGO electrodes without short circuiting at even lower stack pressures (2.5 MPa). When Ag particles were included, the stripping process was similar, but the Ag particles promoted direct deposition of Li within the rGO/Ag scaffold. This uniform deposition of Li contributed to the enhanced cyclability without short circuiting at low stack pressure.

The Li–Ag alloy NPs distributed within the rGO scaffold directed the nucleation and growth of Li within the porous scaffold since Ag tends to chemically alloy with Li to incorporate high Li concentrations. Materials that form solid solutions with Li, such as Ag and Au, support stable cycling due to their homogeneous distribution throughout the scaffold upon stripping. In contrast, materials that form intermetallic compounds, such as Si and Sn in this study, were found to form localized lithiated particles in the composite matrix, which likely cause current concentrations and lead to poor cycling. Overall, our findings demonstrate how different components within Li metal composite electrodes are needed to enhance overall behavior during the stripping and deposition steps, since there are different phenomena that govern behavior during these steps. The Li–Ag alloy and the rGO scaffold act synergistically to effectively deliver Li to the interface, retain electrical contact at the interface, and improve reversibility of morphology changes, resulting in the ability to stably cycle at relatively low stack pressures.

## Methods and Experimental

### Materials

Li_6_PS_5_Cl (LPSC) powders were purchased from MSE supplies (Ampcera) and used as the SSE separator. 25 mg mL^–1^ of concentrated and water-dispersed GO solution (0.5–5 μm flake size, Graphene supermarket) was used for Li/rGO and Li/rGO/Ag electrode fabrication. The GO concentration was diluted to 15 mg mL^–1^ and sonicated for 2 h. In the case of Li/rGO/Ag, Ag nanoparticles (∼150 nm, Sigma-Aldrich) were added to the diluted solution with a mass ratio of 9:1 (GO:Ag) before sonication. The sonicated GO and GO/Ag solutions were spread uniformly on a Teflon plate (∼75 μL cm^–2^) and dried overnight on a 40 °C hot plate. The formed GO and GO/Ag films were thermally reduced at 380 °C on a hot plate in an Ar-filled glovebox, and Li was infiltrated into the rGO and rGO/Ag films by contacting with molten Li.^35^ The fabricated composite foils then went through a successive cold-rolling process to densify the film and control its thickness. The average amount of infiltrated Li in the rGO and rGO/Ag films was 10.29 mg cm^–2^ (±0.87 mg cm^–2^), and the corresponding rGO and Ag mass ratios in the foils were ∼9.9 wt % and ∼1.3 wt %, respectively. The Li/rGO/M electrodes, where M is Au, Si, or Sn nanoparticles (Sigma-Aldrich), were fabricated with identical procedures to the Li/rGO/Ag. Their particle sizes are shown in Table S2.

Li_1_In_3_ alloy counter electrodes were fabricated using an accumulative roll bonding process. In pellets were purchased from Kurt J. Lesker Company. A stacked In/Li/In foil was calendered and folded iteratively until the materials were sufficiently mixed. The atomic ratio of the Li–In alloy was controlled before the initial stacking process, and a 1:3 (Li:In) ratio was used in this study.

Sulfur (S)-multiwalled carbon nanotube (MWCNT)-LPSC composite cathodes were prepared by blending S (100 mesh size, Sigma-Aldrich), MWCNTs (Graphene Supermarket), and LPSC powder (∼1 μm size) with a weight ratio of 2:1:3. A 1.2 g mixture of S, MWCNTs, and LPSC was added into a ZrO_2_ jar with 8 ZrO_2_ balls (diameter: 10 mm). The jar was sealed in an Ar-filled glovebox and blended in a planetary ball mill (Fritsch Pulverisette 7). The mixture was milled with a milling speed of 500 rpm with a total of 24 cycles consisting of alternate milling and resting periods for 10 min each.

### Cell Assembly and Electrochemical Testing

Half-cells used for electrochemical Li stripping tests and EIS analysis were constructed with the composite (or pure Li) electrodes and a Li counter electrode. 90 mg of the LPSC powder (∼10 μm size) was poured into a polyether ether ketone (PEEK) die (diameter: 1 cm) and uniaxially compressed to a pressure of ∼440 MPa for 5 min. The pressed pellets had a typical thickness of 650–700 μm. The Li/rGO, Li/rGO/Ag, or pristine Li foils and Li counter electrode were attached to titanium plungers and inserted to contact both sides of the LPSC pellet. The composite anode/LPSC/Li stack was then uniaxially pressed to 60 MPa to form interfacial contacts. If not specified, the mass of the Li/rGO, Li/rGO/Ag, and Li anode was kept within 8.5–10.2 mg cm^–2^ in the cell assembly. The Li_1_In_3_ alloy electrode served as the counter electrode in the half-cells for the electrochemical cycling tests. The 100–140 μm thick Li_1_In_3_ disk was pressed to the LPSC pellet in the same manner as the half cells for the Li stripping tests.

The S composite electrodes were assembled into Li/rGO/Ag || S full cells. 90 mg of LPSC powder (∼10 μm size) was uniaxially pressed with ∼100 MPa pressure (1 min) in the PEEK die. The S composite powder was then poured on the densified LPSC pellet and uniaxially pressed again at ∼440 MPa for 5 min. After that, Li/rGO/Ag foil was attached on the other side of the LPSC pellet and pressed with ∼60 MPa of pressure. ∼10.2 mg cm^–2^ of the S composite powder was loaded into the cell, which was equivalent to ∼3.4 mg cm^–2^ of the active S in the cells.

The plungers with the cells stacks between them were sandwiched between two steel plates, and the stack pressure was adjusted to match the desired pressure for electrochemical testing (as specified in the main text). The galvanostatic Li stripping and cycling tests (the half-cell tests with Li_1_In_3_ and the Li/rGO/Ag–S full cell tests) were conducted with Arbin and Landt Instruments battery cyclers. The galvanostatic Li stripping with EIS measurement was performed on a Bio-Logic SP-200 potentiostat with frequency range 2 MHz to 2 Hz. Impedance spectra were measured every 0.5 mAh cm^–2^ capacity during the galvanostatic Li stripping. All electrochemical tests were carried out at room temperature (25 °C) in an Ar-filled glovebox.

### Characterization

Scanning electron microscopy (SEM) images were captured with a Zeiss Ultra 60 SEM. The rGO, rGO/Ag, Li/rGO, and Li/rGO/Ag films were transferred into the SEM chamber with a few seconds of exposure to air. 3 or 5 kV of acceleration voltage was used for imaging. Cryogenic focused ion beam (cryo-FIB) SEM imaging was carried out using a Thermo-Fisher Helios 5CX FIB-SEM equipped with a Ga ion source and a Quorum cryogenic stage system. The electrochemically cycled cells were extracted from the PEEK die inside the Ar-filled glovebox and transferred into the SEM chamber with a few seconds of air exposure. All samples were cooled down to −140 °C before the FIB milling and imaging to reduce detrimental interactions with the ion beam.^37^ 65 nA of beam current with 30 kV accelerating voltage was used in the initial cross-section milling. 2.8 nA of beam current was applied for final polishing of the cross-section. Energy dispersive X-ray spectroscopy (EDS) analysis was conducted for elemental mapping and line scanning was conducted with data obtained at 10 kV.

The depth-resolved X-ray photoelectron spectroscopy (XPS) was carried out using a Thermo K-Alpha instrument. Samples were prepared by electrochemically cycling in the cell housings and then disassembling. The samples were transferred to the XPS chamber using a vacuum sealed transfer holder. Measurements were performed with the X-ray beam from an Al Kα source under less than 2.5 × 10^–7^ mbar of chamber pressure. The area from which the signal was obtained was in the middle of the cell and its spot size was 400 μm. Surface charging effects were compensated using a flood gun, and charge referencing was conducted using C 1s (284.7–284.8 eV) for the obtained peaks. The sample surface was etched between each spectrum collection and the etching times were denoted in each graph. In each Ag 3d spectrum, Ag 3d_3/2_ and Ag 3d_5/2_ were observed with a binding energy difference of 6 eV due to spin orbit splitting.^56^

X-ray diffraction (XRD) data was collected using a Rigaku Miniflex 600 with Cu K∝ radiation and 2θ between 10° and 90° with a step size of 0.01°. Each sample was placed on a zero-background sample holder and sealed with Kapton tape in an Ar-filled glovebox.

## Description of the Modeling Framework

### Electrochemical Model

The cell voltage is described as the sum of the kinetic overpotential at the working electrode, ohmic drop in the solid-state electrolyte (SSE), and kinetic overpotential at the counter electrode. For the working electrode that undergoes stripping, the contact fraction affects the kinetic overpotential at the interface and the ohmic response in the SSE (e.g., due to ionic constriction). With respect to the working electrode, the electrochemical reaction and transport have been modeled as follows. The electric potential distribution in the SSE is governed by
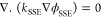
1

Here, *k*_*SSE*_ is the ionic conductivity of the SSE and ϕ_*SSE*_ is the electric potential in response to ionic transport in the SSE. The thickness of the SSE is 700 μm.

The reaction current at the interface is governed by the Butler–Volmer expression. At the contact points,
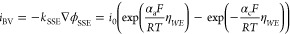
2

Here, *i*_BV_ is the reaction current, *F* is the Faraday constant, *R* is the gas constant, *i*_0_ is the exchange current density, *T* is the temperature, ∝_*a*_ and ∝_*c*_ are the charge transfer coefficients, and η_WE_ is the overpotential of the working electrode. At noncontact points at the interface, *i*_BV_ = 0. The current density (*I*_app_) is applied at the top boundary (i.e., counter electrode), considering complete contact at its interface with the SSE: .

### Correlating Overpotential Response with Interfacial Contact

The cell voltage is a strong function of the contact evolution during stripping. Our electrochemical model allows us to deduce the correlational map between the evolving interface at the anode with the recorded overpotential response. A surface roughness of (*λ*_rms_ = 0.1 μm) is used to generate a stochastic initial interface profile that sets the contact at the start of the simulation at the anode-SSE interface. An empirical relation allows us to effectively vary the contact between the SSE and anode to realize varying contact fraction set points. The mathematical formulation for the surface generation is given below:
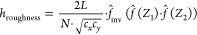
3
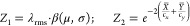
4

Here, *λ*_rms_, *C*_*x*_ and *C*_*y*_ are the roughness parameters of the SSE, *L* is the spatial dimension of the interface (15 μm × 15 μm), *N* is the total number of discrete contact points in the square domain, β(μ,σ) is a random normal distribution of size *Nx1* with mean μ and standard deviation σ,  and  are the x-coordinate and y-coordinate of chosen discrete points in the domain, ,  are the Fourier and inverse Fourier transforms operated on functions *Z*_1_, *Z*_2_ and *h*_roughness_ is the height of the initial roughness profile.

The height of the roughness profile with respect to a reference frame ( = 0) indicates contact/noncontact with the interface and is set to be an initial condition to the electrochemical model. The model further solves for the electric potential distribution inside the SSE based on boundary conditions set by the generated contact profile. The total overpotential is calculated as the sum of interfacial kinetic overpotential and the ohmic drop across the bulk SSE.
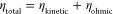
5
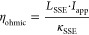
6

Here *η*_kinetic_ is the solution from the electrochemical model dependent on the input conditions like stack pressure (P), current density (*I*_app_), bulk ionic conductivity of the SSE (*K*_SSE_), while *η*_ohmic_ is the fixed ohmic drop calculated based on eq 6 with SSE thickness = 700 μm. Figure S9 shows the correlation between overpotential response and interfacial contact at various current densities ranging from 0.25 to 1 mA cm^–2^.

The contact maps corresponding to the experimental voltage curves during galvanostatic stripping are calculated based on the interfacial contact at the recorded overpotential derived from the correlation map described above. The first step toward estimating the contact fraction is the accurate determination of ohmic drop based on the overpotential value at zero capacity. By fixing the bulk value of ionic conductivity in the SSE, the total overpotential drop can be successfully correlated to the proportionate interfacial contact at the stipulated current. The contact maps generated at a specific capacity (Figure 3c in the manuscript) reaffirm the ability of composite electrodes to enable higher contact retention resulting in lower overpotential of the cell as compared to a standard Li electrode. Pure Li electrodes without a scaffold experience larger polarization resulting in current constriction at isolated contact points further exacerbating the kinetic overpotential drop. Figure S8 shows a steeper drop in contact fraction owing to increased current densities that signal minimal differences in utilization of a scaffold at reaction dominated regimes.

## Supporting Information Available

XPS spectra of rGO film, rGO/Ag film, Ag foil, and lithiated Ag foil; stripped Li capacities and Li usage % (Table S1); Li stripping voltage profiles; Bode plots; Li cycling voltage profiles; size, atomic fraction, and delithiation potentials of the metal nanoparticles (Table S2); Li stripping voltage profiles and corresponding interfacial contact changes; evolution of overpotential response with interfacial contact changes; EDS line scanned profile of delithiated Li/rGO/Ag composite-LPSC interface; electrochemical lithiation profiles of Ag foil and corresponding cross-sectional SEM images; Ag foil lithiation test (Supporting Note 1); EDS line scanned profile and mapping image of Li/rGO/Au and Li/rGO/Si electrodes; electrochemical cycling profiles of Li||S cell (PDF)
