# Supplementary material for: Synergistic Evolution of Alloy Nanoparticles and Carbon in Solid-State Lithium Metal Anode Composites at Low Stack Pressure
Source: ACS Nano. 2024 Jul 29;18(31):20792–805. doi: 10.1021/acsnano.4c07687 (PMC11308923; doi:10.1021/acsnano.4c07687)
Supplement: Supplementary file 1 — nn4c07687_si_001.pdf [file nn4c07687_si_001.pdf]

Supporting Information

Synergistic evolution of alloy nanoparticles and carbon in solid-state lithium metal anode composites at low stack pressure

Sun Geun Yoon[1], Bairav S. Vishnugopi[2], Elif Pınar Alsaç [1], Won Joon Jeong[3], Stephanie Elizabeth Sandoval[1,3], Douglas Lars Nelson[3], Abhinand Ayyaswamy[2], Partha P. Mukherjee[2], and Matthew T. McDowell[1,3]*

1. George W. Woodruff School of Mechanical Engineering, Georgia Institute of Technology, Atlanta, GA 30332, USA

2. School of Mechanical Engineering, Purdue University, West Lafayette, IN 47907, USA

3. School of Materials Science and Engineering, Georgia Institute of Technology, Atlanta, GA 30332, USA

*Corresponding Author: mattmcdowell@gatech.edu

Supporting Figures

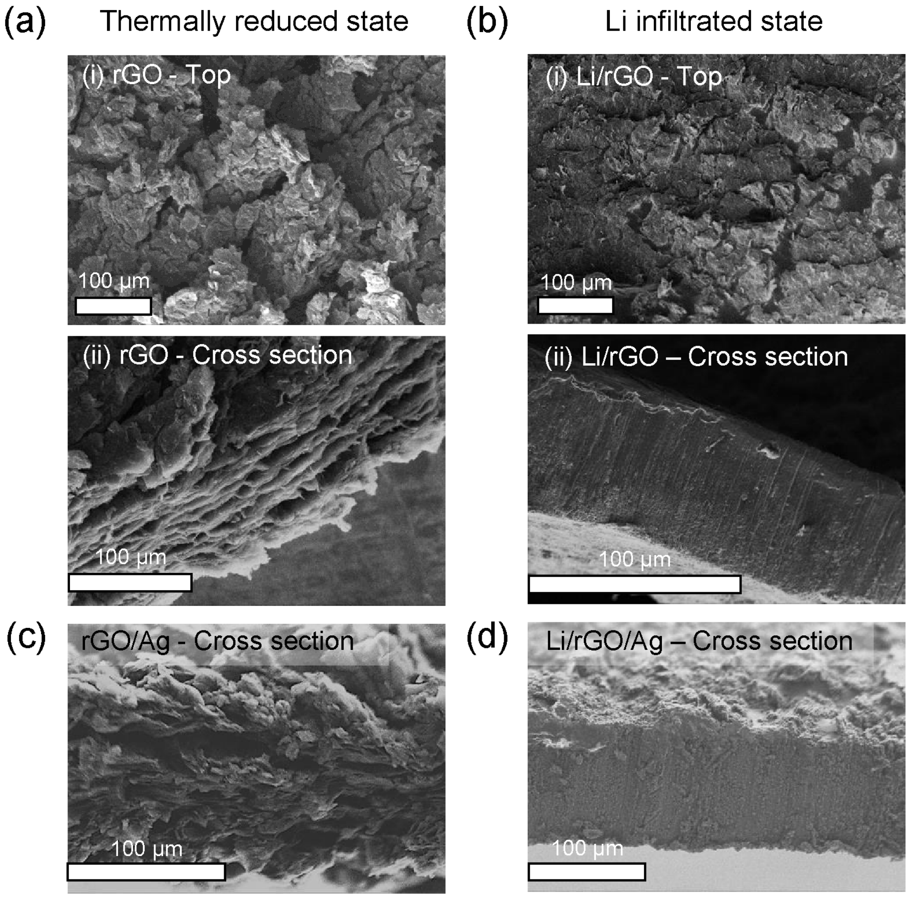

Figure S1. (a-d) SEM images of (a, b) rGO and (c, d) rGO/Ag films after (a, c) thermal reduction and (b, d) Li infiltration.

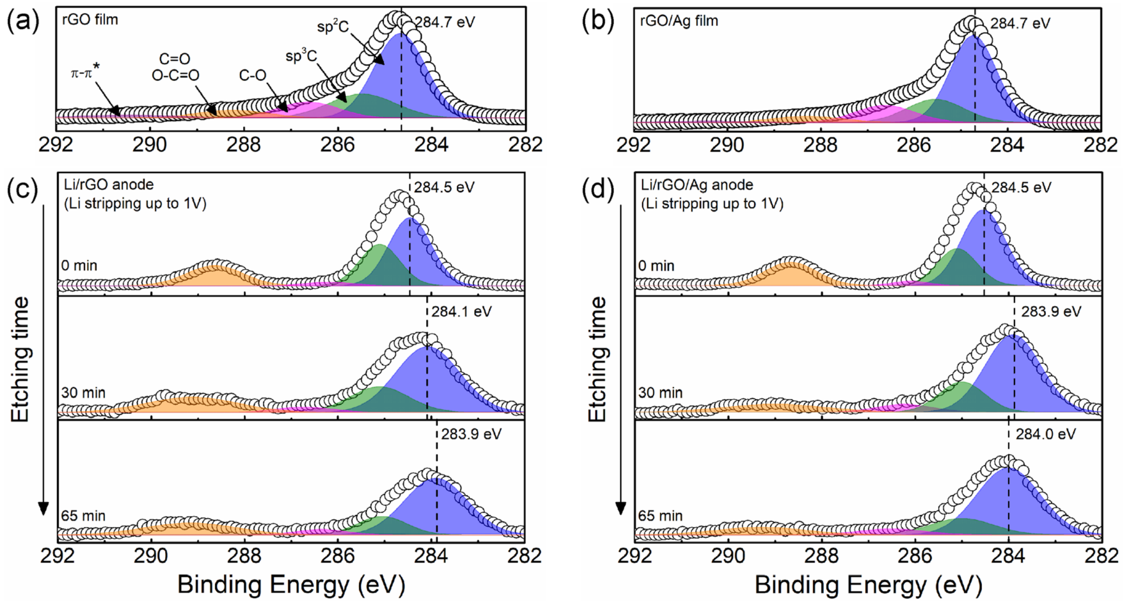

Figure S2. (a, b) XPS C1s spectra of reduced (a) rGO and (b) rGO/Ag films prior to the Li infiltration. Depth-resolved XPS C1s spectra of (c) rGO and (d) rGO/Ag scaffolds on the SSE layer. The Li/rGO and Li/rGO/Ag electrodes were electrochemically stripped up to 1 V cutoff to create these samples. After the Li stripping, Li/rGO- and Li/rGO/Ag-SSE interfaces were easily split in two at the rGO layer, and XPS was used to measure the rGO scaffold surface on the SSE side (as shown in Figures 1e and 5c). Longer etching times indicate a closer position to the SSE layer. The deconvoluted peaks were assigned to each bonding constituent, as shown in (a) [S1,S2]. As compared to the original rGO and rGO/Ag films ((a) and (b)), the major sp^2 C peaks in the Li-infiltrated states ((c) and (d)) were shifted to lower binding energies, which is likely from remaining lithiated carbon (LiC_x) [S3].

Table S1. Anode mass loading, available Li capacity, actual stripped capacity, and Li usage percentage for the tested cells shown in Figures 1b and S3a. Available Li capacities in the Li/rGO and Li/rGO/Ag anodes were calculated by excluding rGO and Ag NPs fractions (9.86 wt. % and 1.3 wt. %, respectively) from the anode mass.

Electrode types	0.25 mA cm^{-2} and 0.8 MPa (Figure 1b)			0.25 mA cm^{-2} and 1.6 MPa (Figure 1b)		
	Anode mass loading [mg] / Available capacity [mAh cm^{-2}]	Stripping capacity at cutoff voltage [mAh cm^{-2}]	Li usage %	Anode mass loading [mg] / Available capacity [mAh cm^{-2}]	Stripping capacity at cutoff voltage [mAh cm^{-2}]	Li usage %
Li	7.7 / 37.9	1.57	4.1%	6.8 / 33.4	11.4	34.1%
Li/rGO	7.6 / 33.2	8.57	25.8%	7.9 / 35.0	22.6	64.6%
Li/rGO/Ag	6.9 / 30.1	10.39	34.5%	6.7 / 29.3	18.2	62.1%

Electrode types	0.25 mA cm^{-2} and 3.2 MPa (Figure 1b)			0.5 mA cm^{-2} and 3.2 MPa (Figure S3b)		
	Anode mass loading [mg] / Available capacity [mAh cm^{-2}]	Stripping capacity at cutoff voltage [mAh cm^{-2}]	Li usage %	Anode mass loading [mg] / Available capacity [mAh cm^{-2}]	Stripping capacity at cutoff voltage [mAh cm^{-2}]	Li usage %
Li	8.0 / 39.3	30.8	78.4	6.5 / 32.0	11.7	36.6
Li/rGO	7.8 / 34.6	28.8	83.2	7.7 / 34.1	23.1	67.7
Li/rGO/Ag	7.2 / 31.4	24.6	78.3	6.8 / 29.7	19.2	64.6

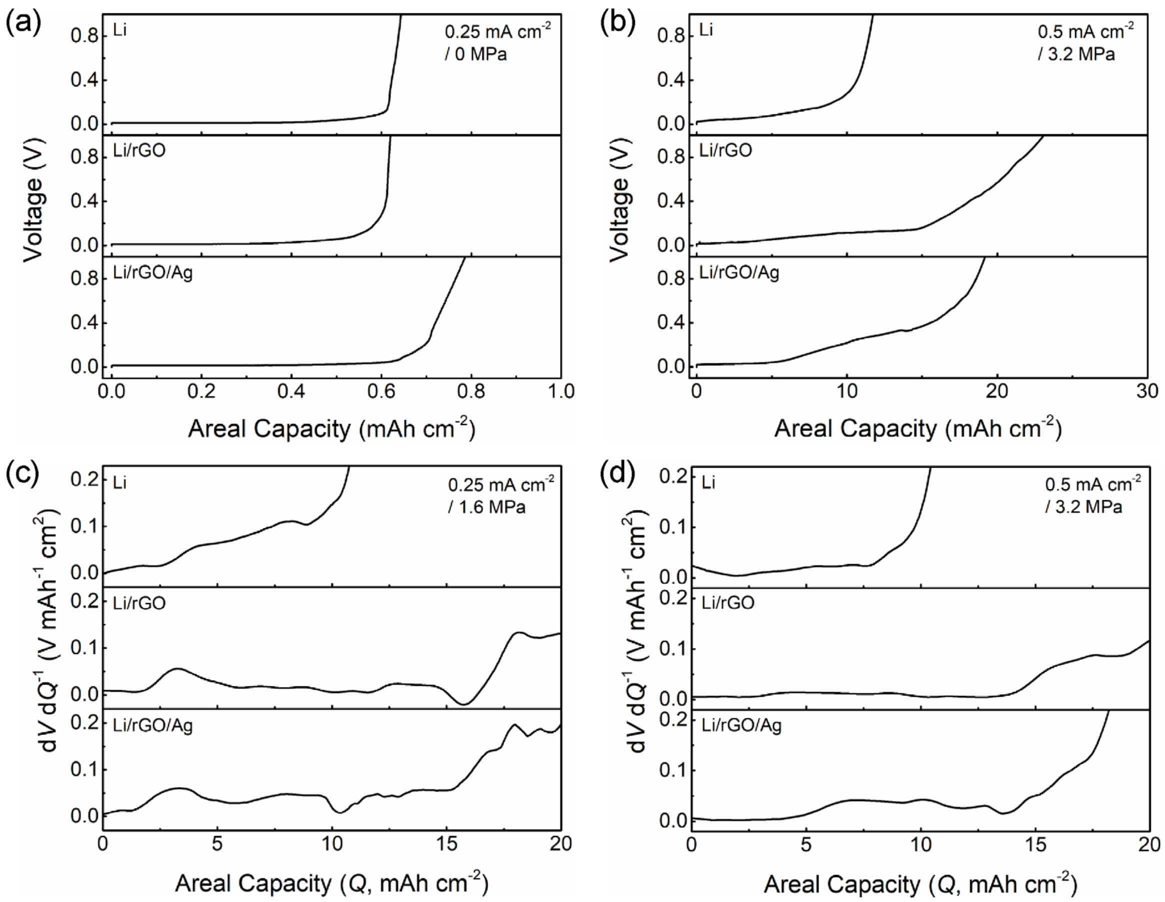

Figure S3. (a, b) Li stripping voltage profiles for Li (top), Li/rGO (middle), and Li/rGO/Ag (bottom) electrodes tested with (a) a current density of 0.25 mA cm^{-2} with 0 MPa stack pressure and (b) a current density of 0.5 mA cm^{-2} and 3.2 MPa stack pressure. The cells were assembled with Li metal counter electrodes. The cells in (a) were subjected to a pressure of approximately 5 kPa from the mass of the Ti plunger and stack plate. (c, d) Differential voltage ($dV\,dQ^{-1}$) profiles for the Li (top), Li/rGO (middle), and Li/rGO/Ag (bottom) electrodes. The plots were derived from the Li stripping voltage curves at 1.6 MPa in Figure 1b and 3.2 MPa in (b). The pure Li electrode in (c) showed gradually increasing $dV\,dQ^{-1}$, indicating steady polarization as the stripping capacity increases. On the other hand, the composite electrodes exhibited relatively flat $dV\,dQ^{-1}$ up to larger areal capacities (14-15 mAh cm^{-2}), implying that polarization was delayed compared to the pure Li.

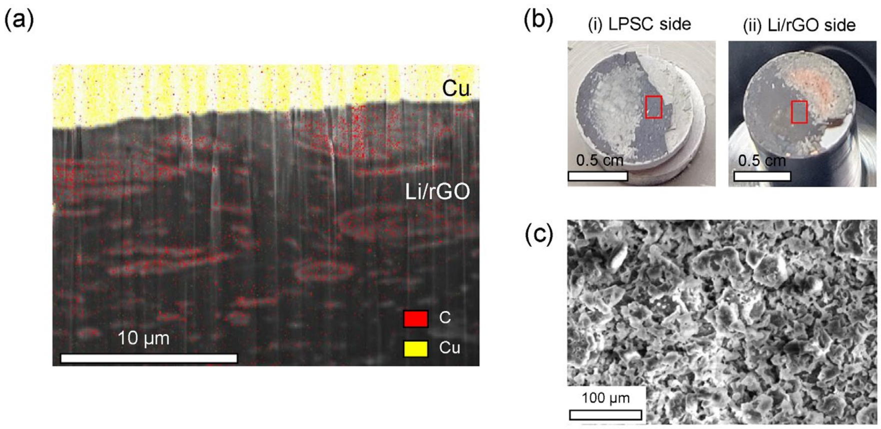

Figure S4. (a) Cryo-FIB-SEM image and overlaid energy dispersive spectroscopy (EDS) elemental map of the pristine Li/rGO anode. (b) Photographs of the Li/rGO|SSE|Li stack after extraction from the PEEK die. The cell underwent Li stripping up to 1 V. After the Li stripping, the Li/rGO anode did not adhere to the SSE, and the Li/rGO-SSE interface was randomly spilt into the rGO scaffold-SSE ((i) LPSC side) and the Li/rGO-rGO scaffold ((ii) Cu side) portions. The red boxed region was ion-beam etched for cross-sectional imaging. (c) An SEM image of the rGO scaffold on the SSE (a top view of (i) LPSC side in Figure 1e).

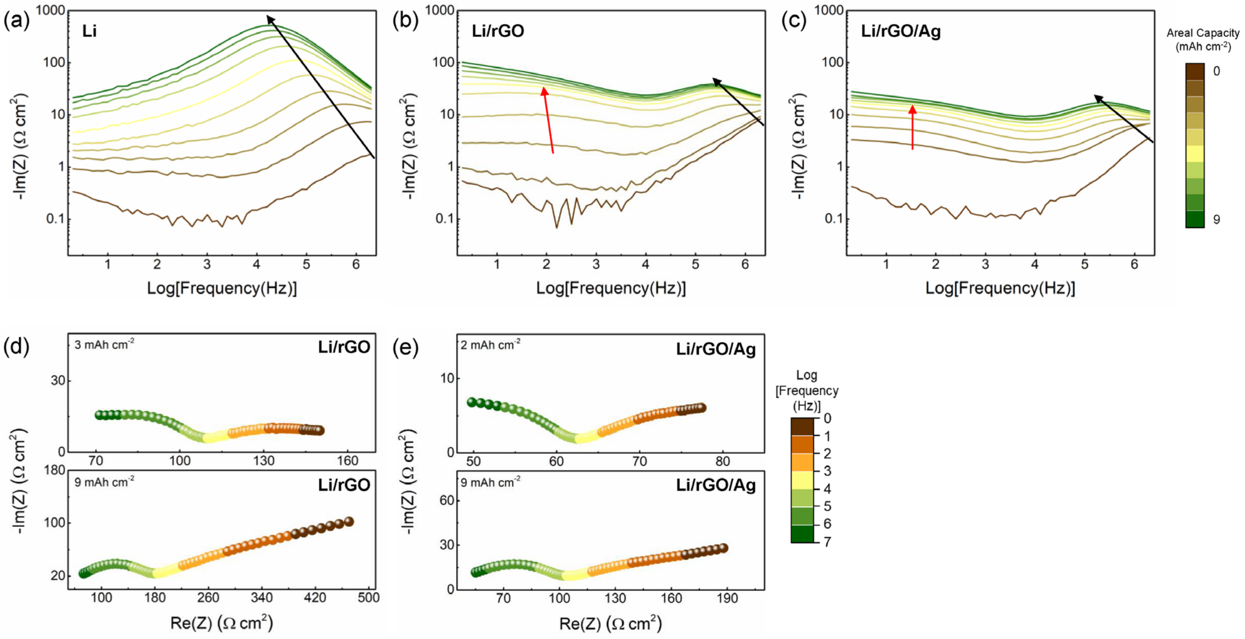

Figure S5. (a-c) Partial Bode plots of (a) Li, (b) Li/rGO, and (c) Li/rGO/Ag electrodes from the stripping tests shown in Figure 2. Black and red arrows in (a-c) denote shifts of apex frequencies by evolution of the semicircles shown in Figures 2b-d. (d, e) Nyquist plots of (d) the Li/rGO electrode at 3 and 9 mAh cm^{-2} stripping capacities and (e) the Li/rGO/Ag electrode at 2 and 9 mAh cm^{-2} stripping capacities. These Nyquist plots in the early and final stages of the Li stripping process are the same as those in Figures 2c, d, but they are shown here at a different scale to aid in visualization.

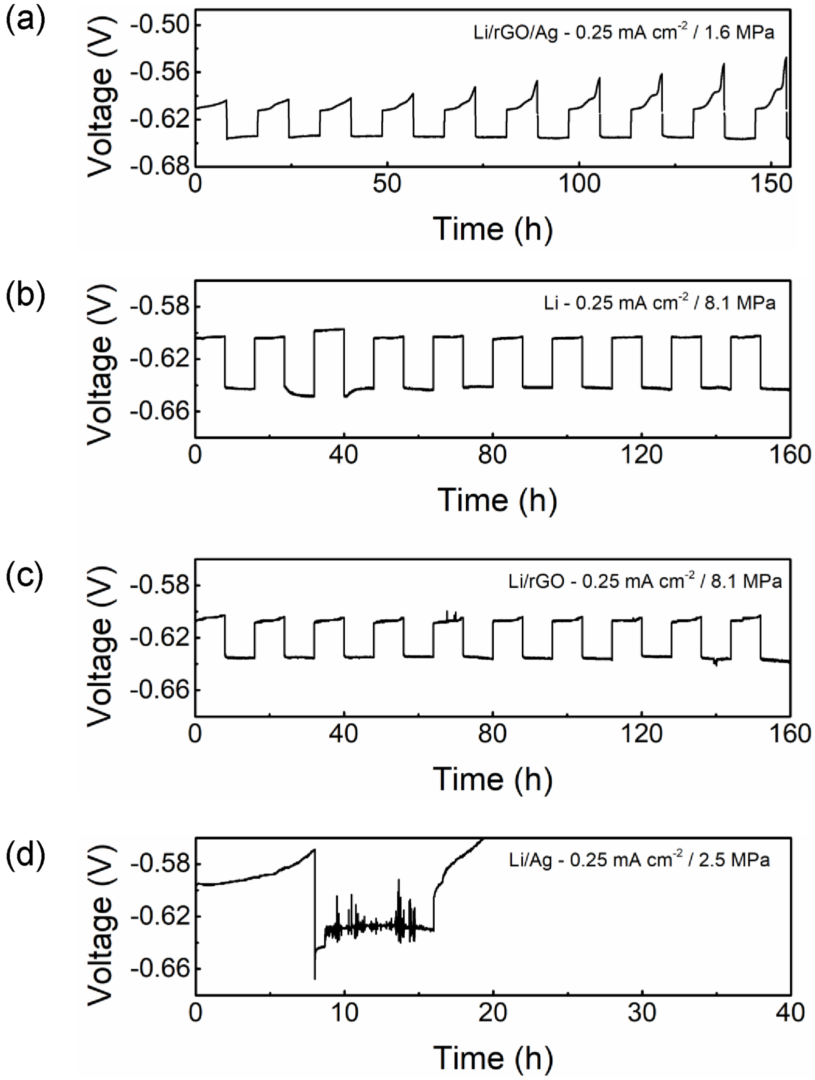

Figure S6. (a-c) Galvanostatic cycling tests of (a) a Li/rGO/Ag electrode at 1.6 MPa stack pressure and (b) Li and (c) Li/rGO electrodes at 8.1 MPa of stack pressure. (d) Galvanostatic cycling test of a Li/Ag electrode without carbon fillers. The cell was tested at 2.5 MPa stack pressure. All the cells in (a-d) were assembled with LiIn counter electrodes (0.62 V *vs.* Li/Li^{+}) and cycled using 0.25 mA cm^{-2} current density and 2 mAh cm^{-2} capacity. The Li/Ag electrode in (d) was created by mixing the same quantity of Ag NPs into molten Li as for the Li/rGO/Ag electrode (~1.3 wt.%).

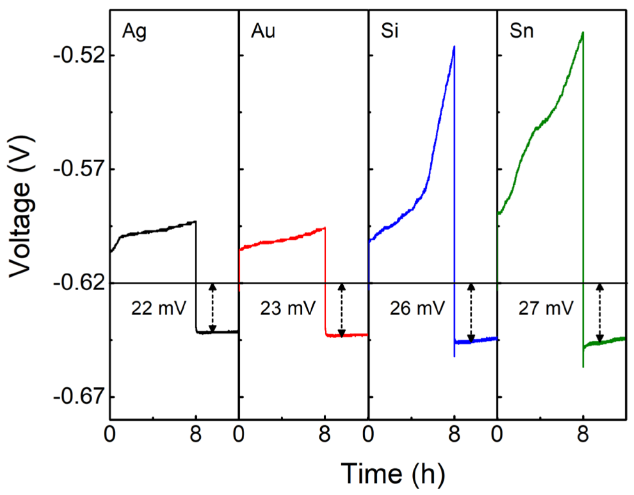

Figure S7. Magnified graphs of the first Li stripping and plating cycles in the Li/rGO/Ag (from Figure 3a), Li/rGO/Au, Li/rGO/Si, and Li/rGO/Sn (from Figure 3d) test results. The Ag and Au composite electrodes had smooth Li plating curves with smaller plateaus (~22 and ~23 mV, respectively), but the Si and Sn composites experienced voltage spikes at the beginning of the plating process, followed by increased plateaus (~26 and 27 mV, respectively).

Table S2. Size, atomic fraction, and delithiation potentials of the metal nanoparticles used in this study. The atomic fraction was calculated based on the mass of nanoparticles loaded into the rGO film and the infiltrated Li in the Li/rGO/M foils (experimental section).

Nanoparticles (M)	Size [nm]	Atomic fraction of M (*vs.* Li in the Li/rGO/M electrode) [at. %]	Delithiation potentials [V *vs.* Li/Li$^+$]	Reference
Ag	<150	0.08	0.04, 0.1	[S4]
			0.02	This study (Figure S12c)
Au	<100	0.04	0.07, 0.16	[S5]
			0.017	[S6]
Si	100	0.31	0.3, 0.49	[S7]
Sn	<150	0.07	0.58, 0.7	[S8]

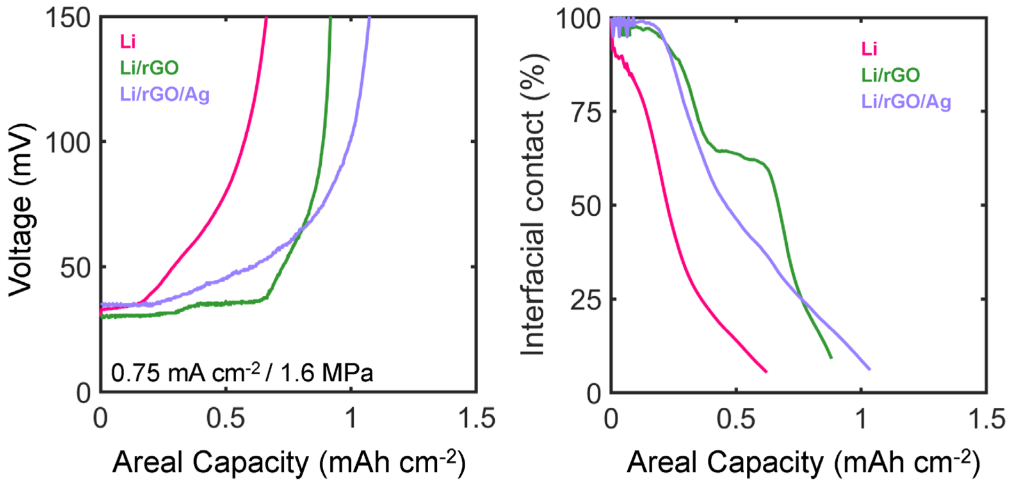

Figure S8. Experimental voltage responses during Li stripping (left panel) and modeled interfacial contact evolution (right panel) of the Li, Li/rGO, and Li/rGO/Ag interfaces for 0.75 mA cm^{-2} current density at 1.6 MPa stack pressure.

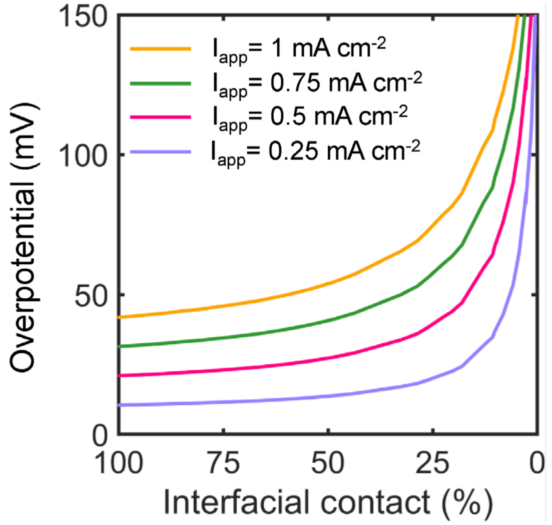

Figure S9. Evolution of overpotential response with interfacial contact from the electrochemical model for various applied currents ranging from 0.25 mA cm^{-2} to 1 mA cm^{-2} current densities at 1.6 MPa stack pressure.

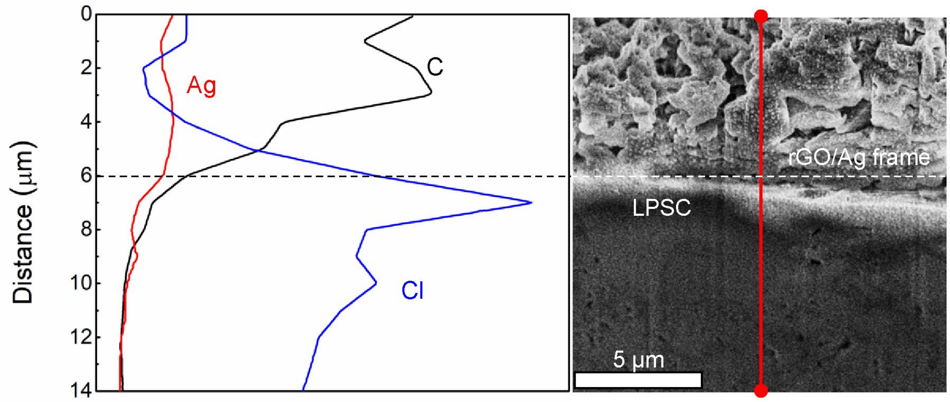

Figure S10. EDS line scan profiles of C, Ag, and Cl at the rGO/Ag scaffold frame-SSE interface (left) and its corresponding position in the cryo-FIB SEM image (right). The SEM image is an enlarged part of the image shown in Figure 5c.

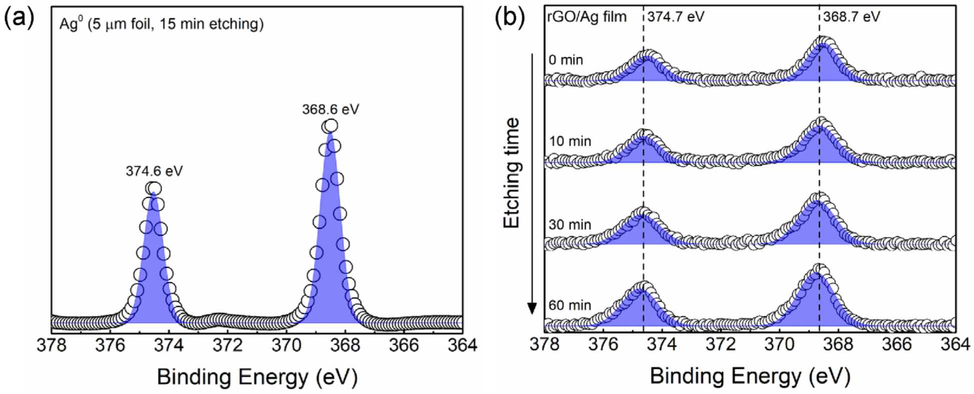

Figure S11. (a) XPS Ag 3d spectra of a pristine Ag foil. Binding energies of Ag^0 were observed at 374.6 (Ag $3d_{3/2}$) and 368.6 (Ag $3d_{5/2}$) eV. (b) XPS depth-resolved Ag 3d spectra of the rGO/Ag film prior to Li infiltration. Binding energies of the Ag NPs were similar to the Ag^0 result, and Ag NPs were uniformly distributed in the rGO film with negligible chemical state changes.

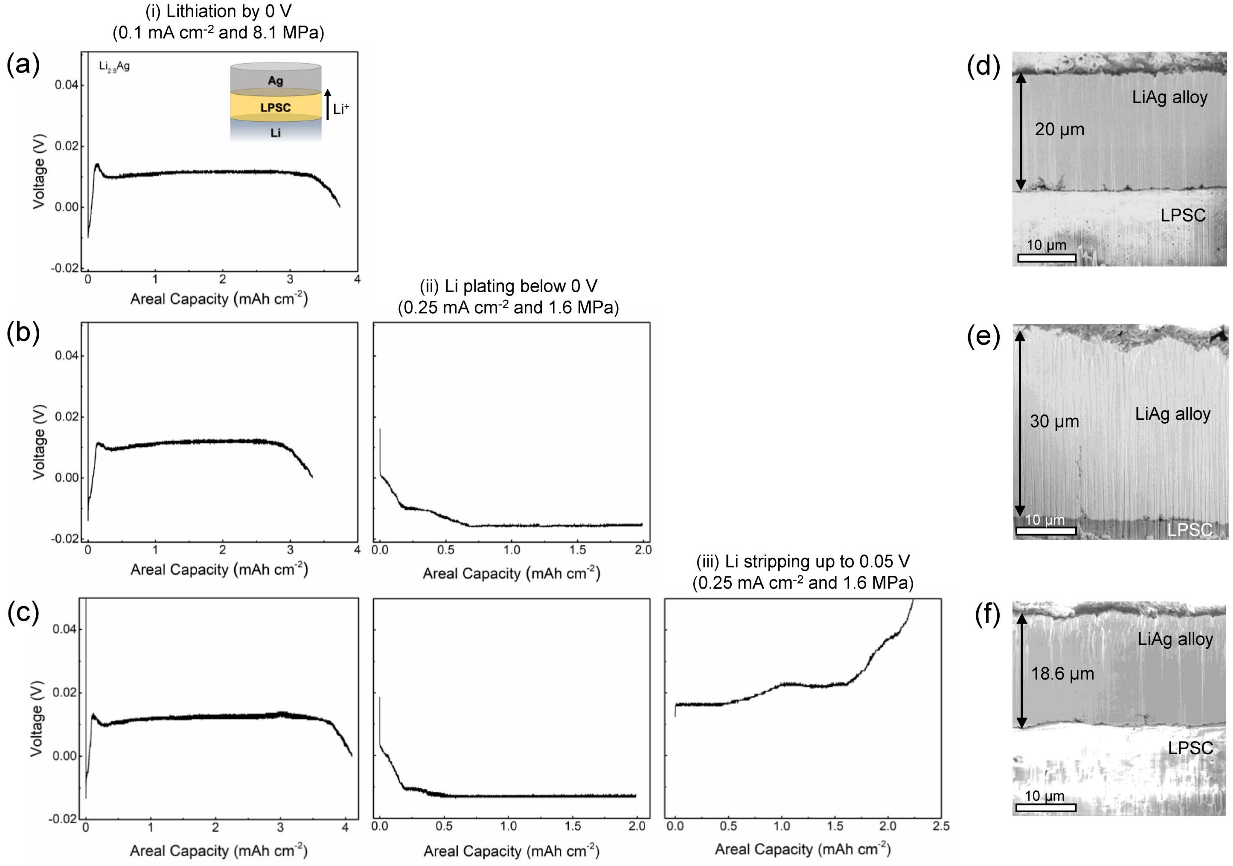

Figure S12. Electrochemical lithiation and cycling of Ag foil electrodes along with cryo-FIB SEM images at different states. (a) Voltage profile of a Ag anode showing (i) lithiation to 0 V after an initial nucleation overpotential, and a schematic image of the constructed cell. (b) Voltage profiles of a Ag anode showing (i) lithiation until 0 V followed by (ii) 2 mAh cm^{-2} Li plating of the same electrode. (c) Voltage profiles of a Ag anode showing (i) lithiation until 0 V, (ii) subsequent 2 mAh cm^{-2} Li plating, and then (iii) Li stripping up to 0.05 V. Cryo-FIB SEM images of the Ag electrode-SSE interface (d) after the electrochemical lithiation shown in (a), (e) after the lithiation and Li plating shown in (b), and (f) after the electrochemical lithiation, Li plating, and Li stripping/delithiation shown in (c). The first lithiation steps of all Ag anodes (process (i)) were conducted with 0.1 mA cm^{-2} current density at 8.1 MPa of stack pressure. The Li cycling on the lithiated Ag anodes (processes (ii) and (iii)) was carried out at 0.25 mA cm^{-2} and 1.6 MPa of current density and stack pressure, respectively, similar to the Li/rGO/Ag electrode cycling test conditions. The Ag electrodes were controlled to have a thickness of ~5 μm, and deviation of the foil thickness might cause lithiation capacity differences as shown in the lithiation curves ((i) in (a-c)). Based on the lithiation capacity shown in (a), a stoichiometric ratio of the LiAg alloy in (d) was represented as $Li_{2.9}Ag$ in this study. More information on these experiments is provided in Supporting Note 1.

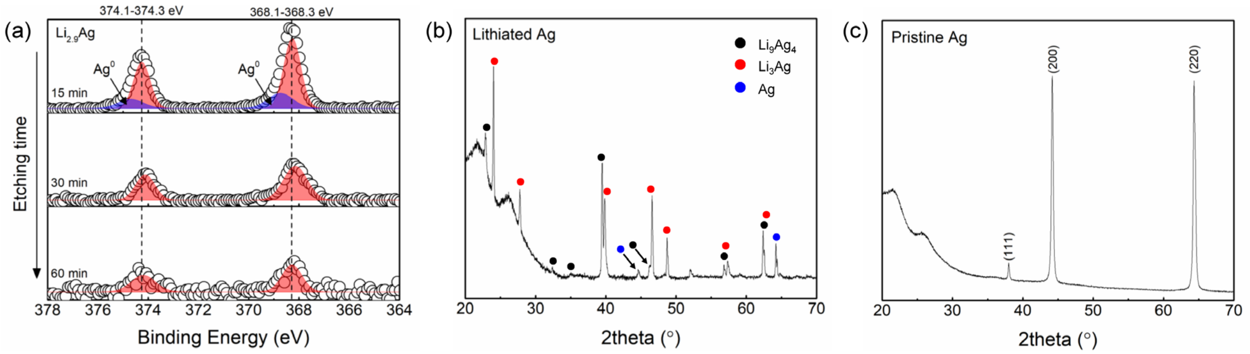

Figure S13. (a) Depth-resolved Ag 3d XPS spectra of the lithiated Ag anode shown in Figure S12a, d. The longer etching time indicates a closer position to SSE. The Ag $3d_{3/2}$ and Ag $3d_{5/2}$ peaks did not show meaningful position changes with etching time. Only the Ag^0 shoulders at 15 min etching time were observed, which might come from less lithiation due to being at the farthest position from the SSE. (b) X-ray diffraction pattern of electrochemically lithiated Ag shown in Figure S12a, d. (c) X-ray diffraction pattern of pristine Ag foil (5 μm thickness). The diffraction patterns are analyzed based on a database at the Materials Project as follows; Li_9Ag_4 (mp-1222549), Li_3Ag (mp-865875), and Ag (mp-124) [S9]. The broad peak between 20° and 30° came from the Kapton tape window.

Supporting Note 1. Ag foil lithiation test

Ag foils (~5 μm thick) were used to investigate (de)lithiation behaviors of Ag. Half-cells were assembled with the Ag foil working electrode and Li counter electrode as shown in the inset image of Figure S12a. The three different LiAg alloying states were prepared by electrochemical lithiation/delithiation and their cross-section images were captured using cryo-FIB SEM as follows.

(i) Lithiation of the pristine Ag foil to a cutoff voltage of 0 V. The cells were operated with 8.1 MPa of stack pressure and the Ag anode was lithiated using a current density of 0.1 mA cm^{-2} for stable alloying.

(ii) After conducting step (i), 2 mAh cm^{-2} of Li was further plated on the sample using a current density of 0.25 mA cm^{-2} at 1.6 MPa stack pressure.

(iii) After conducting (i) and (ii), Li was stripped from the alloy foil to a cutoff of 0.05 V using a current density of 0.25 mA cm^{-2} at 1.6 MPa stack pressure. The cutoff voltage of 0.05 V was set to monitor delithiation behavior near the Li redox potential (0 V *vs.* Li/Li^{+}).

Electrochemical voltage profiles and corresponding cross-sectional images of the samples after steps (i), (ii), and (ii) are shown in Figures S12a, d, Figure S12b, e, and Figure S12c, f, respectively. As Li plating can occur below 0 V, the alloying state (i) was set as a transition point to distinguish the sole process of Li alloying from the combined Li plating and alloying processes. The alloying state (i) was characterized with XPS and XRD, as shown in Figure S13a, b, and its binding energy positions were utilized to reference the chemical states of Ag NPs in the delithiated rGO/Ag sample (Figure 6b). To mimic the Li/rGO/Ag testing condition shown in Figure 3a, lithiation/delithiation after the transition point (the alloying states (ii) and (iii)) were conducted with 0.25 mA cm^{-2} at 1.6 MPa of stack pressure.

After the lithiation above 0 V (stage (i)), the Ag foil became almost four times thicker than its initial thickness (~5 μm) (Figure S12d). A stoichiometric ratio of $Li_{2.9}Ag$ was obtained from the lithiation voltage profile of the sample (i) (3.74 mAh cm^{-2}, Figure S12a). The XRD characterization (Figure S13b) shows that the sample (i) has Li-rich alloy phases, such as Li_3Ag and Li_9Ag_4, which were similar to the electrochemical stoichiometric ratio. The samples after stages (ii) and (iii) also featured capacities of 3.33 and 4.10 mAh cm^{-2}, respectively (Figure S12b, c), during the initial lithiation (the alloying process (i)). These capacities were similar to the lithiation capacity in the Li alloying state (i) (Figure S12a). After further lithiation below 0 V (sample (ii)), the alloy foil became 1.5 times thicker (~30 μm) than sample (i) without visible Li metal in the sample. As shown in Figure S12b, the early-stage voltage profile was smooth without any nucleation overpotentials, and two voltage plateaus, a shorter one at -10 mV followed by a longer one at -15 mV, were

observed during the lithiation (Figure S12b). These two plateaus could represent electrochemical alloying of and then Li plating on the Ag foil since they are less than the Li redox potential (0 V *vs.* Li/Li^+) [S6,S10]. However, after observing the two voltage plateaus in the electrochemical lithiation, the foil having only a homogeneous phase was captured as shown in Figure S12e. This indicated that, even after the electrochemical process ended, the deposited Li could dissolve spontaneously into the alloy foil, or the lithiated Ag alloy phases could be homogenized. This result is possibly responsible for high Li diffusion coefficient in LiAg alloy [S10], and a thermodynamic driving force for this spontaneous alloying was investigated by Kim *et al.* [S4]. The alloying state (iii) was achieved by delithiating 2.23 mAh cm^{-2} of Li from sample (ii), resulting in the foil thickness being reduced by 18.6 µm. As shown in Figure S12c, the electrochemical profiles displayed multiple plateaus even below 0.05 V, which could include Li stripping and dealloying processes. After the delithiation, the foil was preserved without any cracks or voids (Figure S12f).

The series of electrochemical lithiation profiles and the corresponding *ex situ* cross-sectional imaging suggest the following points.

1. After passing the transition state (x>2.9 in Li_xAg), the lithiation/delithiation of the LiAg alloy can occur along with Li plating and stripping due to the similar alloying potential and Li redox potential (0 V *vs.* Li/Li^+) [S6,S10].

2. The lithiation of the LiAg alloy foil over the transition state showed negligible energy barriers for Li alloying and plating. In addition, the deposited Li was spontaneously dissolved into the alloy foil. These results demonstrate a highly favorable LiAg alloying process for stoichiometries richer in Li than $Li_{2.9}Ag$.

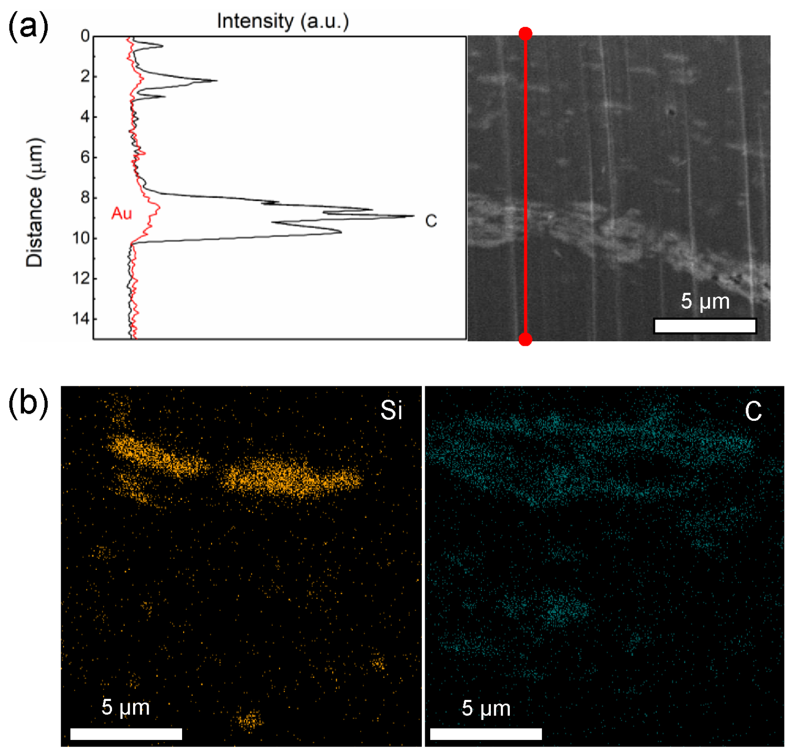

Figure S14. (a) EDS line scan profiles of C and Au in the Li/rGO/Au anode and its corresponding line position in the cryo-FIB SEM image. The SEM image is an enlarged part of the image shown in the red box of Figure 5d. (b) EDS elemental maps of Si (left) and C (right) from the Li/rGO/Si anode shown in the red box of Figure 5e.

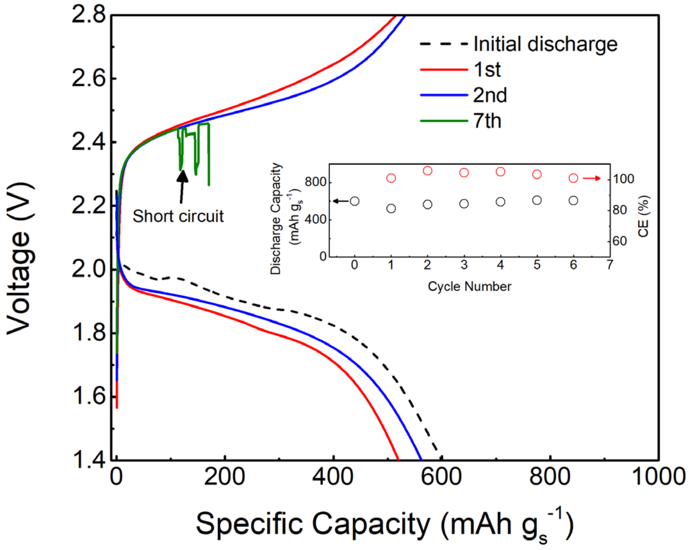

Figure S15. Electrochemical cycling test of a full cell with pure Li anode and sulfur-based cathode at 4.9 MPa stack pressure. The cell was discharged on the first cycle at 0.1 mA cm^{-2} to activate the S cathode and then cycled between 1.4 and 2.8 V using 0.25 mA cm^{-2} current density. 3.4 mg cm^{-2} of sulfur was loaded into the cell, which is equivalent to 5.7 mAh cm^{-2} theoretical capacity. The inset graph shows discharge capacity and cycling efficiency of the cell. The testing temperature is 25 °C. The cell was short circuited after six cycles.

Supporting Information References

[S1] Chen, X.; Wang, X.; Fang, D. A review on C1s XPS-spectra for some kinds of carbon materials. *Fuller. Nanotub. Carbon Nanostructures* **2020**, *28*, 1048-1058. DOI: 10.1080/1536383X.2020.1794851.

[S2] Högström, K.C.; Malmgren, S.; Hahlin, M.; Gorgoi, M.; Nyholm, L.; Rensmo, H.; and Edström, K. The buried carbon/solid electrolyte interphase in Li-ion batteries studied by hard X-ray photoelectron spectroscopy. *Electrochim. Acta* **2014**, *138*, 430-436. DOI: 10.1016/j.electacta.2014.06.129.

[S3] Chen, H.; Yang, Y.; Boyle, D.T.; Jeong, Y.K.; Xu, R.; de Vasconcelos, L.S.; Huang, Z.; Wang, H.; Wang, H.; Huang, W.; Li, H.; Wang, J.; Gu, H.; Matsumoto, R.; Motohashi, K.; Nakayama, Y.; Zhao, K.; Cui, Y. Free-standing ultrathin lithium metal–graphene oxide host foils with controllable thickness for lithium batteries. *Nat. Energy* **2021**, *6*, 790-798. DOI: 10.1038/s41560-021-00833-6.

[S4] Kim, S.Y.; Li, J. Porous mixed ionic electronic conductor interlayers for solid-state batteries. *Energy Mater. Adv.* **2021**, *2021*, 1519569. DOI: 10.34133/2021/1519569.

[S5] Sandoval, S.E.; Lewis, J.A.; Vishnugopi, B.S.; Nelson, D.L.; Schneider, M.M.; Cortes, F.J.Q.; Matthews, C.M.; Watt, J.; Tian, M.; Shevchenko, P.; Mukherjee, P.P.; McDowell, M.T. Structural and electrochemical evolution of alloy interfacial layers in anode-free solid-state batteries. *Joule* **2023**, *7*, 2054-2073. DOI: 10.1016/j.joule.2023.07.022.

[S6] Zhang, S.; Yang, G.; Liu, Z.; Weng, S.; Li, X.; Wang, X.; Gao, Y.; Wang, Z.; Chen, L. Phase diagram determined lithium plating/stripping behaviors on lithiophilic substrates. *ACS Energy Lett.* **2021**, *6*, 4118-4126. DOI: 10.1021/acsenergylett.1c02127.

[S7] Yao, Y.; McDowell, M.T.; Ryu, I.; Wu, H.; Liu, N.; Hu, L.; Nix, W. D.; Cui, Y. Interconnected silicon hollow nanospheres for lithium-ion battery anodes with long cycle life. *Nano Lett.* **2011**, *11*, 2949-2954. DOI: 10.1021/nl201470j.

[S8] Courtney, I.A.; Tse, J.; Mao, O.; Hafner, J.; Dahn, J.R. Ab initio calculation of the lithium-tin voltage profile. *Phys. Rev. B* **1998**, *58*, 15583. DOI: 10.1103/PhysRevB.58.15583.

[S9] Data retrieved from the Materials Project for Li_9Ag_4 (mp-1222549), Li_3Ag (mp-865875), and Ag (mp-124) from database version v2023.11.1.

[S10] Jin, S.; Ye, Y.; Niu, Y.; Xu, Y.; Jin, H.; Wang, J.; Sun, Z.; Cao, A.; Wu, X.; Luo, Y.; Ji, H.; Wan, L.-J. Solid–solution-based metal alloy phase for highly reversible lithium metal anode. *J. Am. Chem. Soc.* **2020**, *142*, 8818-8826. DOI: 10.1021/jacs.0c01811.
